# Identification and Analysis of Novel Viral and Host Dysregulated MicroRNAs in Variant Pseudorabies Virus-Infected PK15 Cells

**DOI:** 10.1371/journal.pone.0151546

**Published:** 2016-03-21

**Authors:** Fei Liu, Hao Zheng, Wu Tong, Guo-Xin Li, Qing Tian, Chao Liang, Li-Wei Li, Xu-Chen Zheng, Guang-Zhi Tong

**Affiliations:** 1 Department of Swine Infectious Diseases, Shanghai Veterinary Research Institute, Chinese Academy of Agricultural Sciences, Shanghai, People’s Republic of China; 2 Jiangsu Co-innovation Center for Prevention and Control of Important Animal Infectious Diseases and Zoonoses, Yangzhou, Jiangsu, People’s Republic of China; Institut Pasteur of Shanghai, Chinese Academy of Sciences, CHINA

## Abstract

Pseudorabies (PR) is one of the most devastating diseases in the pig industry. To identify changes in microRNA (miRNA) expression and post-transcriptional regulatory responses to PRV infection in porcine kidney epithelial (PK15) cells, we sequenced a small RNA (sRNA) library prepared from infected PK15 cells and compared it to a library prepared from uninfected cells using Illumina deep sequencing. Here we found 25 novel viral miRNAs by high-throughput sequencing and 20 of these miRNAs were confirmed through stem-loop RT-qPCR. Intriguingly, unlike the usual miRNAs encoded by the α-herpesviruses, which are found clustered in the large latency transcript (LLT), these novel viral miRNAs are throughout the PRV genome like β-herpesviruses. Viral miRNAs are predicted to target multiple genes and form a complex regulatory network. GO analysis on host targets of viral miRNAs were involved in complex cellular processes, including the metabolic pathway, biological regulation, stimulus response, signaling process and immune response. Moreover, 13 host miRNAs were expressed with significant difference after infection with PRV: 8 miRNAs were up-regulated and 5 miRNAs were down-regulated, which may affect viral replication in host cell. Our results provided new insight into the characteristic of miRNAs in response to PRV infection, which is significant for further study of these miRNAs function.

## Introduction

Pseudorabies virus (PRV) belongs to the family *Herpesviridae* and is the causative agent of Aujeszky’s disease. PRV causes neurological and respiratory system disorders in newborn piglets and reproductive disease in pregnant sows [1]. The widely used PRV Bartha-K61 strain-based vaccine has played an important role in the control and eradication of PR worldwide. In late 2011, however, PRV variant outbreaks among Bartha-K61-vaccinated pigs in China. The PRV variant JS-2012 strain was isolated from a Bartha-K61-vaccinated pig farm in Jiangsu Province of China in 2012 [2, 3]. This variant caused higher mortality than did a previously isolated classic virulent PRV strain, SC. The Bartha-K61 did not fully protect piglets against challenge with the variant JS-2012 strain [3]. This new PRV variant that has emerged in PR vaccine-immunized swine populations in China has caused significant economic losses to the domestic swine industry [4–7].

miRNAs are small ssRNA species (~20–24 nucleotides) that regulate mRNA expression through post-transcriptional mechanisms [8]. Mature miRNAs are incorporated into the RNA-induced silencing complex (RISC), leading to either mRNA degradation or translational repression [8]. miRNAs can modulate, to varying degrees, the expression of hundreds of different target genes, including genes that regulate immunity, apoptosis and key steps in the virus life cycle such as latency, lytic infection and the transition from latency to lysis [9, 10].

As with many other alphaherpesviruses, PRV expresses multiple viral miRNAs [11–13] in multiple cells and tissues. Eleven PRV miRNAs have been identified in previous studies [14–16]. Since even single nucleotide changes can dramatically alter miRNA specificity, miRNAs are prone to accelerated evolution, especially in viruses [17, 18]. Here we constructed and compared sRNA libraries prepared from PRV JS-2012-infected and uninfected PK15 cells using Illumina deep sequencing, both to identify viral miRNAs and to characterize cellular miRNAs for their potential roles in the host response to variant PRV infection. Our findings described the PRV virus-host interaction at the overall miRNA level. These data may accelerate the understanding of PRV pathogenesis and other herpesviruses.

## Materials and Methods

### Viruses and cells

The PRV variant JS-2012 strain (GenBank NO. KP257591), the classic virulent PRV SC and vaccine Bartha-K61 strain were kept in our laboratory [3, 19]. PK15 cells purchased from ATCC (Manassas, VA, USA) were used for virus propagation in Dulbecco’s Modified Eagle’s Medium (DMEM) supplemented with 10% fetal bovine serum (Invitrogen, Carlsbad, CA, USA).

### RNA extraction

PK15 cells (3×10^6^ cells per flask) were infected with PRV at a multiplicity of infection (MOI) of 0.01 and incubated at 37°C for 1 h with rocking every 15 min. Cell monolayers were washed with phosphate-buffered saline (PBS) and fresh medium was added to the infected cells. Cells were harvested for RNA extraction at 2, 4, 6, 8, 12, 18, 24, 30 and 36 h post-infection (pi). The total RNA was extracted from PK15 cells using the miRNeasy Mini Kit (QIAGEN) and genomic DNA was removed using the RNase-Free DNase Set (QIAGEN). RNA (5 μL) extracted from the nine time points was mixed and quantified using a NanoDrop 2000 Spectrophotometer (Thermo). The mixed RNA quality was assessed using an Agilent 2100 Bioanalyzer. sRNA sequencing was further performed using the Illumina Genome Analyzer (Illumina, Huada Genomics Institute Co. Ltd, China).

### Stem-loop RT-qPCR assay

Assays to quantify the mature miRNAs were conducted as previously described [20]. Briefly, 10 ng of total RNA was reverse-transcribed to cDNA by using the RevettAid^tm^ Frist Strand cDNA Synthesis Kit (Thermo) together with miRNA specific stem-loop RT (S1 Table). Samples were incubated at 42°C for 1 h and at 70°C for 5 min. Next, PCR was performed for each miRNA using the transcription product and miRNA specific forward primer and universal reverse primer (S1 Table). Real-time PCR was performed using a SYBR Premix *Ex Taq™* (Takara) in an Eppendorf Mastercycler ep realplex (Germany). In each assay, 2 μL cDNA was used for amplification. The amplification conditions were as follows: 95°C for 1 min, followed by 40 cycles at 95°C for 15 s and 64°C for 30 s. miRNA expression was normalized to U6 small nuclear RNA (snRNA). The relative quantitative miRNA expression level was evaluated using the comparative Ct method [20]. Relative expression was calculated by the comparative ΔΔCt method, and the values were expressed as 2^-ΔΔ Ct^.

### Analysis of virally encoded miRNA

The sRNA tags were aligned to the known PRV miRNA precursor in miRBase 21.0 (http://www.mirbase.org/) with no mismatch and then aligned to the corresponding mature miRNA with at least 16 nts overlap allowing offsets. The known viral miRNAs of PRV JS-2012 included the miRNA precursor, length and count of tags would be obtained. Mireap software (http://sourceforge.net/projects/mireap/) was used to predict virally encoded novel miRNA by exploring the secondary structure, Dicer cleavage sites and predicted minimum free energies of unannotated sRNA tags which could be mapped to genome.

### Target prediction of viral miRNAs and GO analysis

Target genes were predicted for virally encoded miRNA using RNAhybrid and miRanda software. Complying with the following criteria in seed region: 1) no mismatch between 1–9 nts on the 5’ end; 2) G-U is permitted, but the number cannot exceeds 3. The Gene Ontology (GO) program Blast2GO (https://www.blast2go.com/) was used to annotate potential host target genes to create histograms of GO annotation, including cell component, biological process and molecular function. GO enrichment analysis of the target genes was performed using PANTHER Classification System [21] to detect the significantly enriched GO terms of the host target genes compared to the Sus scrofa genome-wide background.

### Analysis for host miRNAs

The known and novel host miRNAs in JS-2012-infected and uninfected samples were analyzed and compared. Relative miRNA abundance levels were normalized to the number of transcripts per million (TPM) in each sample. Normalization formula: Normalized expression = Actual miRNA count/Total count of clean reads*1000000. A difference of at least 2-fold between libraries was considered significant for further analysis.

### Sources of genome sequences

The PRV JS-2012, a new virulent pandemic strain, was used for infection and subsequent analysis [2]. JS-2012 full genome has been sequenced and is 143,461 bp long (GenBank NO. KP257591). Sus scrofa genome assembly (Sscrofa10.2) has been produced by the International Swine Genome Sequencing Consortium and 3’ UTR of pig genes were retrieved from Ensembl database (www.ensembl.org).

## Results

### Analysis of sRNAs libraries from solexa sequencing

**Fig 1:**
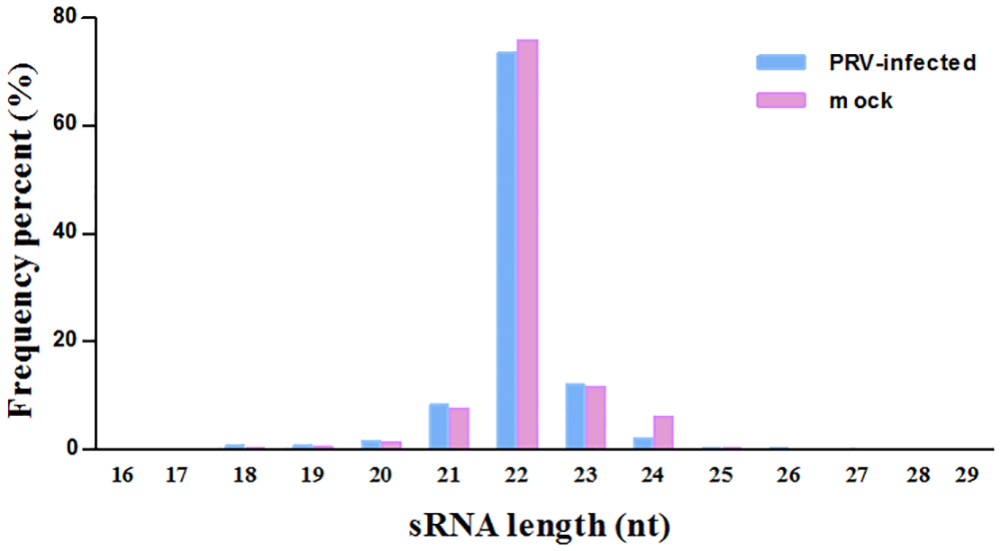
Length distributions of sRNAs (18–30 nt) in PRV-infected and uninfected PK15 cells.

A total of sRNAs reads 114,766,884 and 111,468,414 of 18 to 30 nucleotides in length were obtained from PRV JS-2012-infected and uninfected PK15 cells, respectively. After removing low quality reads and masking adaptor sequences, 112,037,904 (97.62%) and 109,595,539 (98.32%) clean sRNA reads were obtained from the two sRNAs libraries, respectively. Within each sample, 97.81% and 98.75% high quality sRNAs were 20–24 nts in length, with most sRNAs 22 nts in length (Fig 1). The sRNAs were categorized and annotated by following the priority rule: rRNA (in which Genbank > Rfam) > known miRNA > repeat > exon > intron (Table 1). Ultimately, 87,409,488 (78.02%) and 88,667,800 (80.9%) miRNA reads from two libraries were matched to known PRV and host miRNAs sequences, and then 19309634 (17.23%) and 16515597 (15.07%) unannotated sRNA reads from two libraries were matched to PRV and pig genome for predicting novel miRNA.

**Table 1:** Distribution of sRNAs in PRV-infected and uninfected samples.

Category	PRV-infected				Uninfected			
	Unique	(%)	Total	(%)	Unique	(%)	Total	(%)
**Total**	1,035,394	100	112,037,904	100	971,825	100	109,595,539	100
**Exon antisense**	683	0.07	1,117	0	789	0.08	1,209	0
**Exon sense**	8,734	0.84	16,589	0.01	13,138	1.35	22,309	0.02
**Intron antisense**	23,353	2.26	210,710	0.19	30,741	3.16	193,569	0.18
**intron sense**	174,548	16.86	1,334,446	1.19	220,826	22.72	1,197,915	1.09
**miRNA**	4,581	0.44	87,409,488	78.02	4,489	0.46	88,667,800	80.9
**rRNA**	36,478	3.52	1,196,939	1.07	32,388	3.33	963,096	0.88
**repeat**	37,020	3.58	432,889	0.39	43,406	4.47	642,224	0.59
**scRNA**	502	0.05	1,059,031	0.95	486	0.05	489,317	0.45
**snRNA**	6,010	0.58	186,756	0.17	5,809	0.6	126,485	0.12
**snoRNA**	4,323	0.42	96,049	0.09	3,770	0.39	57,162	0.05
**srpRNA**	252	0.02	1,171	0	254	0.03	1,119	0
**tRNA**	61,638	5.95	783,085	0.7	64,533	6.64	717,737	0.65
**Unannotated RNA**	677,272	65.41	19,309,634	17.23	551,196	56.72	16,515,597	15.07

### Stem-loop RT-qPCR for miRNAs detection

**Fig 2:**
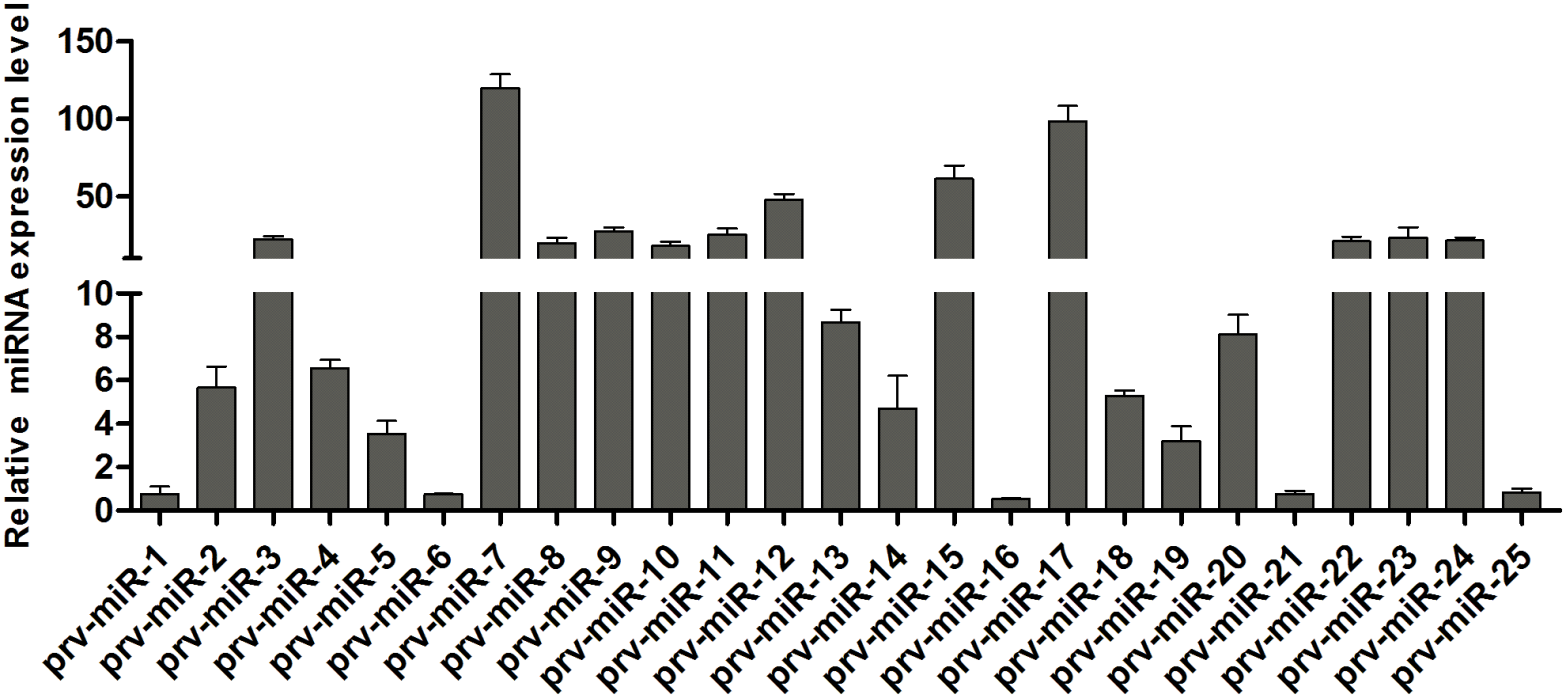
Expression levels detection of virally encoded novel miRNAs using stem-loop qRT-PCR. miRNAs expression levels were normalized to the U6 snRNA. Data are shown as the average ± standard deviation from three independent experiments.

**Fig 3:**
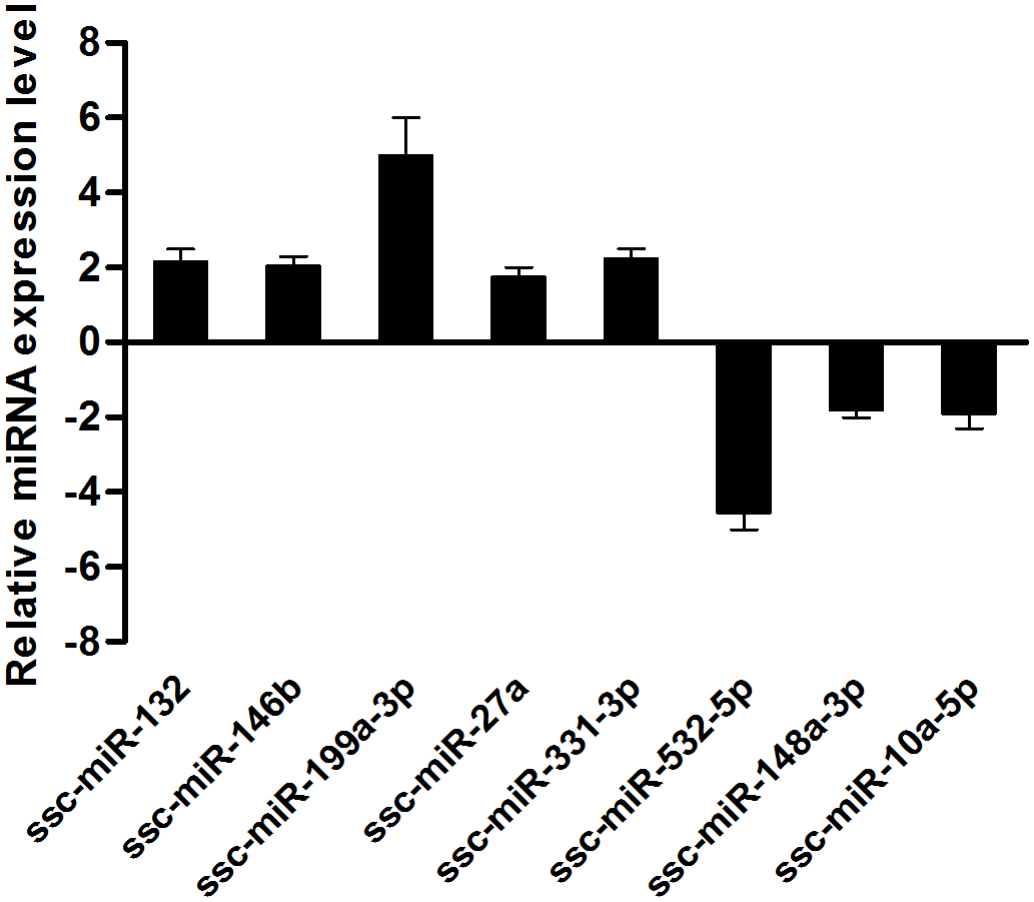
Validation of differentially expressed host miRNAs using stem-loop RT-qPCR. miRNAs expression levels were normalized to the U6 snRNA. The experiments were performed with at least three independent experiments.

To validate the deep sequencing results, stem-loop RT-qPCR was used to verify the expression of virally encoded candidate miRNAs. Among 25 mature viral miRNAs, 20 miRNAs expressed in PRV JS-2012-infected PK15 cells were detected using this approach, with the exception of prv-miR-1, 6, 16, 21 and 25 (Fig 2). Stem-loop RT-qPCR was also used to confirm the differentially expressed host miRNAs. Eight differentially expressed miRNAs were analyzed (Fig 3). Overall, the relative expression levels of these miRNAs detected with RT-qPCR were consistent with the relative expression levels determined with deep sequencing.

### Analysis of virally encoded miRNA

All 11 known mature miRNA sequences in PRV JS-2012 infected sample were detected and then compared with previous studies [14] (Table 2). The dominant sequences of prv-miR-LLT1-3p, prv-miR-LLT3-3p, prv-miR-LLT4-5p, prv-miR-LLT10a/b-3p and prv-miR-LLT11a/b-5p were identical, while the prv-miR-LLT2-5p, prv-miR-LLT5-3p, prv-miR-LLT6-5p, prv-miR-LLT7-5p, prv-miR-LLT8-5p and prv-miR-LLT9-5p sequences differed slightly as compared with the miRNAs described in miRBase. Mostly, several bases were added in the 3’ end, while a base changed in the 5’ end of prv-miR-LLT5-3p (insertion) and prv-miR-LLT9-5p (deletion). In general, the 11 known PRV miRNAs were comparatively conservative and highly expressed in PK15 cells infected with the variant JS-2012 strain.

**Table 2:** The differences (D) or similarities (S) between the known viral miRNAs from JS-2012 strain and miRBase.

miRNA	Sequence(5’-3’)	Length	Strand and Position	Reads
prv-miR-LLT1-3p (S)	TCTCACCCCTGGGTCCGTCGC ^a^	21	+98550–98570	148492
prv-miR-LLT2-5p (D)	CTCATCCCGTCAGACCTGCGCC ^a^	22	+99014–99035	1141
	CTCATCCCGTCAGACCTGCG^b^	20	+99014–99033	284
prv-miR-LLT3-3p (S)	CGCACACGCCCCTCTCGCGCAC ^a^	22	+99114–99135	987
prv-miR-LLT4-5p (S)	AGAGTATCAGCGTGGCTTTTTT ^a^	22	+99394–99415	19472
prv-miR-LLT5-3p (D)	ATGAGTGGATGGATGGAGGCGA ^a^	22	+100603–100624	21596
	TGAGTGGATGGATGGAGGCGAG^b^	22	+100604–100625	17690
prv-miR-LLT6-5p (D)	CGTACCGACCCGCCTACCAGGCA ^a^	23	+100238–100260	7457
	CGTACCGACCCGCCTACCAGG^b^	21	+100238–100258	64
prv-miR-LLT7-5p (D)	CCGGGGGGTTGATGGGGATGGG ^a^	22	+99979–100000	4181
	CCGGGGGGTTGATGGGGAT^b^	19	+99979–99997	41
prv-miR-LLT8-5p (D)	GTGGGGGCGAAGATTGGGTTGGG ^a^	23	+100912–100934	12911
	GTGGGGGCGAAGATTGGGTT^b^	20	+100912–100931	377
prv-miR-LLT9-5p (D)	TCGAGGAGATGTGGAGGGGTGC ^a^	22	+101067–101088	23870
	ATCGAGGAGATGTGGAGGGG^b^	20	+101066–101085	265
prv-miR-LLT10a/b-3p (S)	CCGAGCCTGCCCCTTCCGTCGCA ^a^	23	+102570-102592/-144577-144599	1894
prv-miR-LLT11a/b-5p (S)	AGGCTGGGAGTGGGGACGGAAGA ^a^	23	+102707-102729/-144440-144462	30780

^a^: The most abundant viral miRNAs sequences in this study.

^b^: The viral miRNAs sequences in this study were the same as the miRBase.

**Fig 4:**
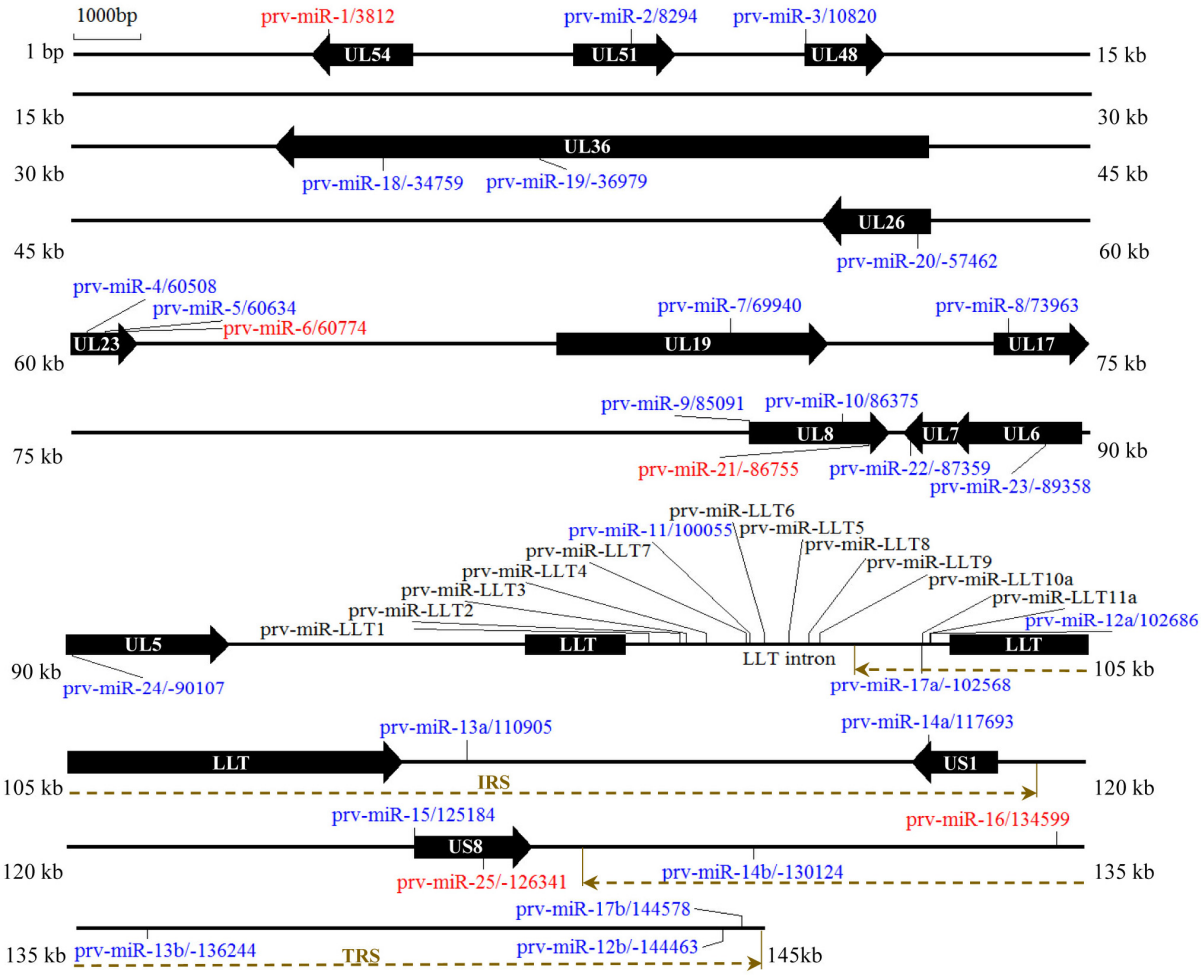
Location of virally encoded miRNA in the PRV JS-2012 genome. The relative positions of the known and predicted novel miRNAs in the JS-2012 genome are shown. The linear form indicated PRV JS-2012 genome. The black arrows indicated predicted miRNAs locations in ORFs of PRV JS-2012. The known miRNAs were indicated with black font. Validated putative miRNAs were indicated with blue font and non-validated putative miRNAs were indicated with red font. The figure showed only ORFs contained the viral miRNAs, virus others genes and 5’ or 3’ UTRs, DNA repeats, splice sites, and origin of replication were not shown.

Twenty-five new candidate viral miRNAs were predicted using Mireap software (Table 3). The energetically stable hairpin structures of 25 novel viral miRNAs and their expression profile were listed in S1 File. Three novel miRNAs, prv-miR-11-5p, prv-miR-12a-3p, prv-miR-17a-5p were located in the LLT region, while the other miRNAs were distributed throughout the PRV genome. Some miRNAs were presented in two copies, including-miR-LLT10a/b-3p, prv-miR-LLT11a/b-5p, prv-miR-12a/b-3p, prv-miR-13a/b-5p, prv-miR-14a/b-5p and prv-miR-17a/b-5p. miRNAs located in the internal repeat sequence (IRS) were designated as ‘a’ and miRNAs in terminal repeat sequences (TRS) were designated as ‘b’ (Fig 4). Moreover, the prv-miR-18 through prv-miR-25, prv-miR-12b, prv-miR-13b, prv-miR-14b and prv-miR-17a were encoded on the opposite DNA strand to the latency associated transcript. This phenomenon appears to be a common feature in the herpesviruses (Fig 4).

**Table 3:** Summary of virally encoded novel miRNA.

miRNA	Sequence(5’-3’)	Length	Strand and Position	Reads	RT-qPCR
prv-miR-1-5p	GGCGGTCGGGGGGCGCGTCGGGC	23	+3812–3834	478	-
prv-miR-2-3p	CATGCACCTGTACCTCTCGG	20	+8294–8313	136	+
prv-miR-3-3p	CGGCCAGCCCGGACGCGCTGTA	22	+10820–10841	5	+
prv-miR-4-3p	CGACGACTGGGGGCGCGCGCC	21	+60508–60528	16	+
prv-miR-5-3p	CGAGCTCTGCGACCGGCGCG	20	+60634–60653	10	+
prv-miR-6-5p	CGCAGGCGCGCGGCATGGAGGT	22	+60774–60795	10	-
prv-miR-7-5p	TACCCGGCGCCCGTGAACGCGCTC	24	+69940–69963	5	+
prv-miR-8-3p	CGAGCTCCTGCCGGCCCGCACG	22	+73963–73984	9	+
prv-miR-9-5p	TACGCGGCGCGCTTCGTCCACG	22	+85091–85112	6	+
prv-miR-10-5p	CCCGCGGACGCGCCGGAGGCGG	22	+86375–86396	105	+
prv-miR-11-5p	TCCACTTCTCGACGGCCGGCTCC	23	+100055–100077	28	+
prv-miR-12a/b-3p	CCAATCGGGTGGCAGCGGGGG	21	+102686-102706/-144463-144483	400	+
prv-miR-13a/b-5p	CCGGGGAAGGGTCGGGCGATG	21	+110905-110925/-136244-136264	983	+
prv-miR-14a/b-5p	CGCCTCGGGGCCGACGTGAACC	22	+117693-117714/-130124-130145	39	+
prv-miR-15-5p	TCTCCGCCGAGACGACCCCGGGC	23	+125184–125206	7	+
prv-miR-16-3p	CGCGGGCGGCGGGAGGGGAGGGA	23	+134599–134621	96	-
prv-miR-17a/b-5p	GCGACGGAAGGGGCAGGCTCGGCG	24	+144578-144601/-102568-102591	5635	+
prv-miR-18-3p	GGCCGGAACACCGAGCGATGG	21	-34759-34779	6	+
prv-miR-19-5p	ACGGAGCGCCTGGACGCCGCGGC	23	-36979-37001	103	+
prv-miR-20-3p	CCGTGCTGGCCGTGGTCGACGA	22	-57462-57483	114	+
prv-miR-21-3p	CACGCGGCGGGGGCGAGGGCGTCC	24	-86755-86778	7	-
prv-miR-22-3p	CCCGACGGGCTGGTGCGGACG	21	-87359-87379	105	+
prv-miR-23-5p	CTGGATCGTGTGCCTCGGCGTG	22	-89358-89379	11	+
prv-miR-24-3p	CGTTGAGGGTCTGGATGCACG	21	-90107-90127	34	+
prv-miR-25-5p	ACGGGCGCGCGGGCGGCGCCGG	22	-126341-126362	5	-

This table showed the name, sequences, length, genomic position and cloning frequency of novel miRNAs encoded by JS-2012 strain in this analysis. The qPCR results showed whether each PRV miRNA was detected (+) or not (-).

### Prediction targets of virally encoded miRNA and functional analysis

**Fig 5:**
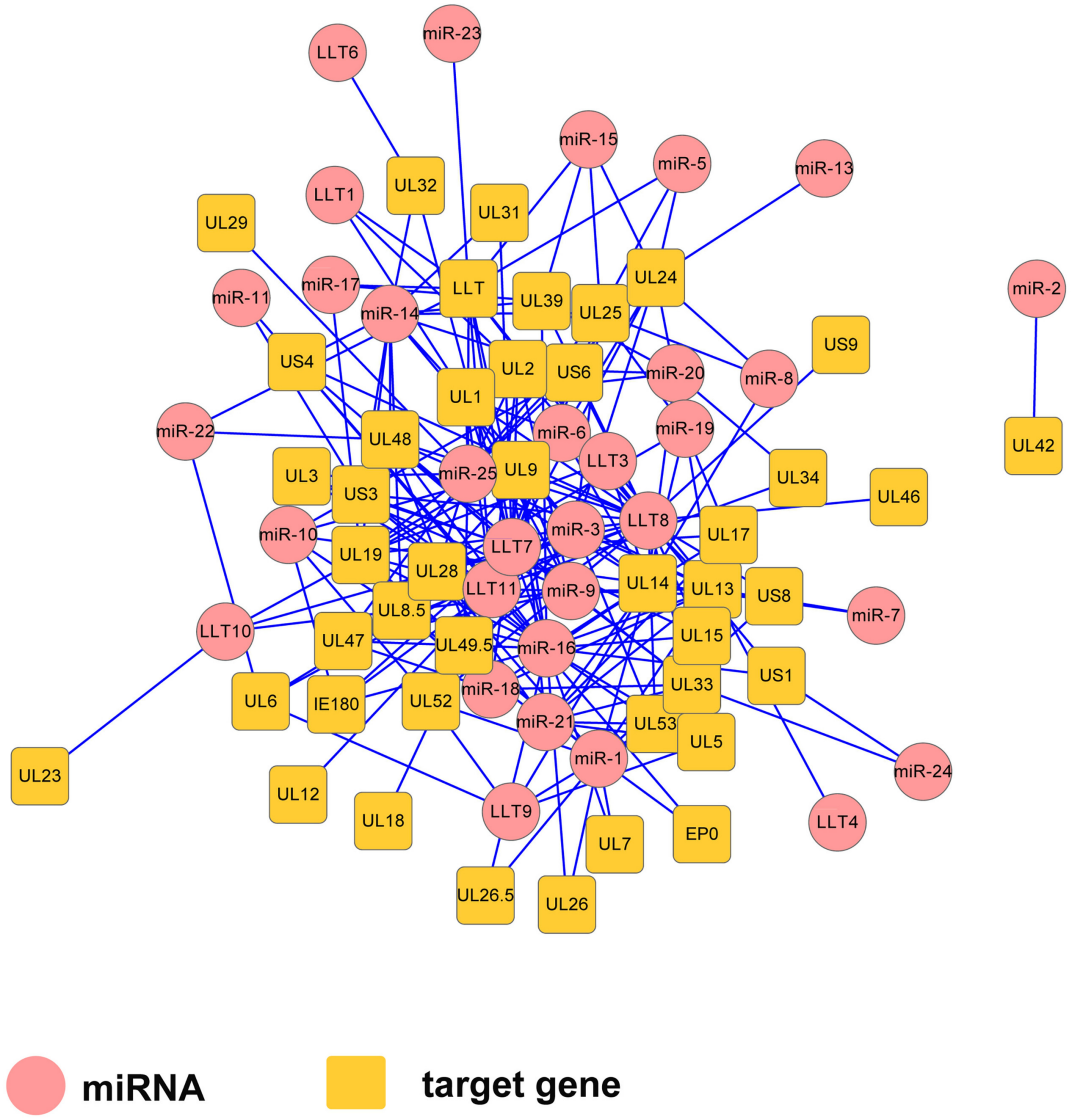
Gene regulatory network formed by PRV JS-2012-encoded miRNAs (circles) and their target genes (rectangles).

A total of 36 miRNAs, including 11 known miRNAs and 25 novel miRNAs, were mapped to the PRV JS-2012 genome. We used RNAhybrid and miRanda software to scan the potential viral gene targets for these miRNAs. We scanned the 3’UTRs of PRV-encoded genes, including the LLT and found that each gene can be targeted by one or more miRNAs. Each of miRNAs are predicted to target multiple genes and form a complex regulatory network (Fig 5). The results revealed the complexity interaction network formed by viral miRNAs and their targets. Notably, prv-miR-1, prv-miR-14a, prv-miR-17a, prv-miR-21, prv-miR-24 and prv-miR-25 were encoded directly antisense to the individually corresponding coding gene, which could theoretically lead to the cleavage of the transcript and negative regulation of the gene.

**Fig 6:**
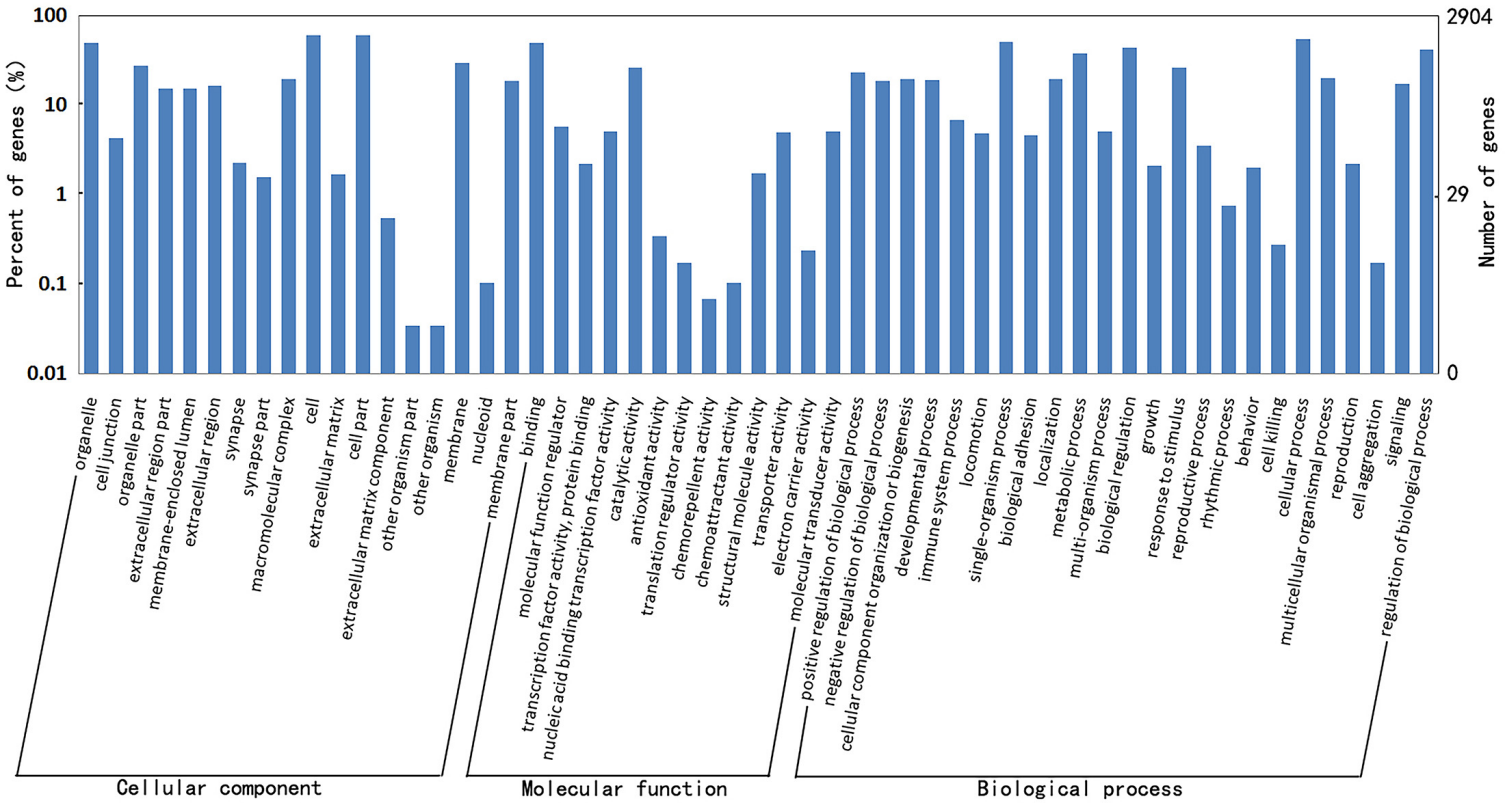
GO annotation on host targets of the viral miRNAs. The figure showed the GO annotation of these targets in biological processes, cellular components and molecular functions.

The potential host targets of 36 viral miRNAs were predicted by RNAhybrid and miRanda software. We found thousands of putative targets combined two programs (S2 Table). In order to investigate biological function of these host targets, GO annotation was performed for the putative host targets. Results revealed that these host targets belonged to the following categories: biological regulation, stimulus response, metabolic process and cellular process, which were similar to the previous study [14]. Besides, the new functions of these newly viral miRNAs were found in this study, such as signal-organism process and signaling process and others (Fig 6). Furthermore, GO enrichment analysis showed that these host targets were functionally enriched in regulation of biological process, immune system process, apoptosis, cell death and others (P < 0.05, S3 Table). These results revealed the complexity of interaction network formed by viral miRNAs and their host targets.

### Expression and differential analysis for host miRNAs

**Fig 7:**
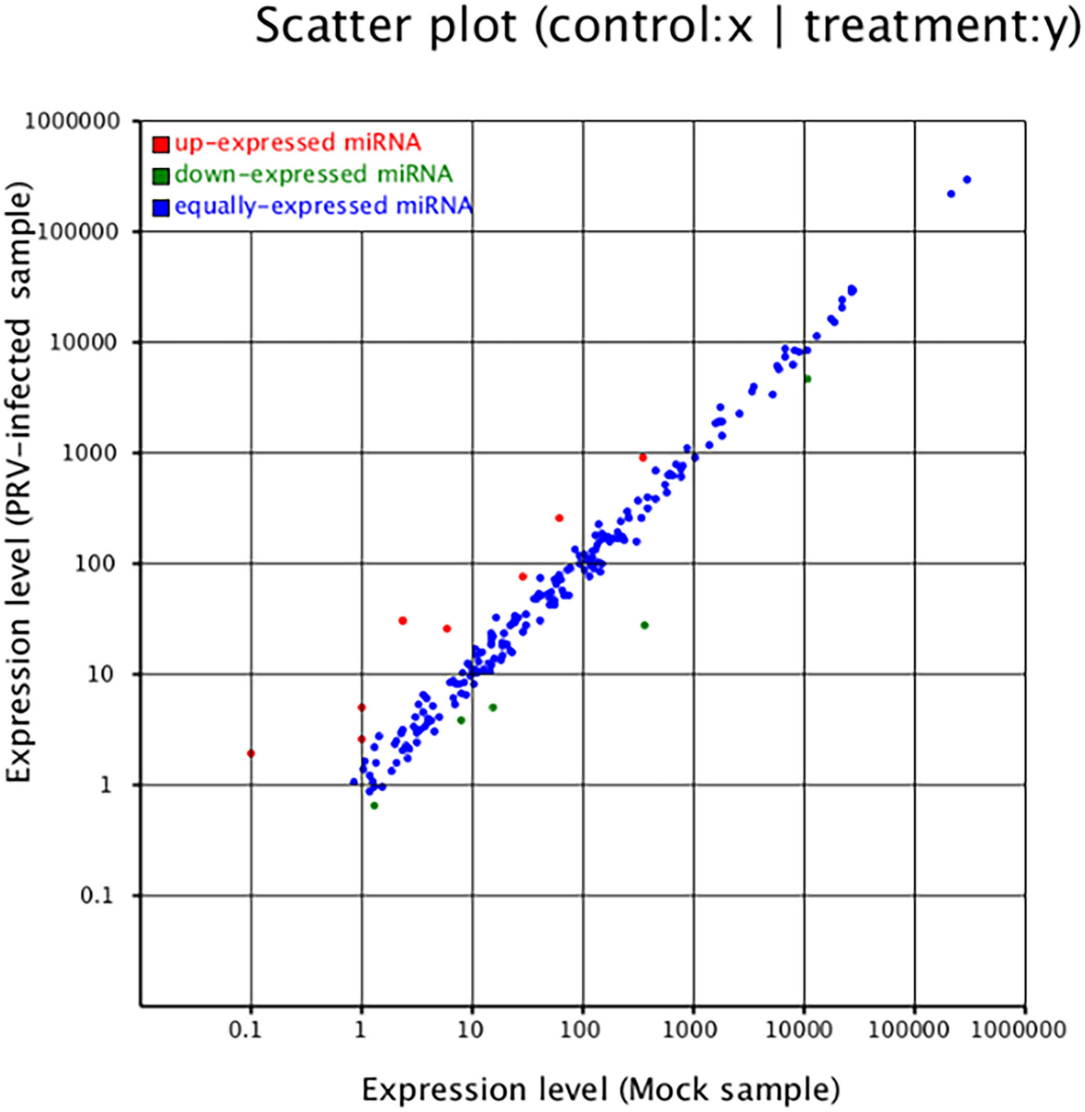
Differential expression of host miRNAs as a function of PRV JS-2012 infection. Red, miRNAs with ratio > 2 (infected/uninfected in expression); blue, miRNAs with 1/2 ≤ ratio ≤ 2; green, ratio < 1/2.

By mapping the clean reads to the miRBase and Sus scrofa genome, 598 host miRNAs were detected in this study. Among of these, 283 known and 239 novel (S4 Table) were found in both libraries, 44 miRNAs were found only in the PRV-infected group and 32 miRNAs were unique to the PRV-uninfected group. In both libraries, the 15 most abundant miRNAs accounted for 92.65% and 92.32% of the total miRNA reads in the infected and uninfected samples, respectively (Table 4). Among the 15 most abundant miRNAs, the most strongly expressed miRNA in both libraries was ssc-let-7 (f, a, d, e, g, c), which represented 74.98% and 74.39% of the total miRNA reads in two samples, respectively. This is consistent with previous reports that ssc-let-7 is highly expressed in various cell types and species [22]. Moreover, 13 miRNAs were differentially expressed: 8 were upregulated (ssc-miR-132, ssc-miR-146b, ssc-miR-215, ssc-miR-371, ssc-miR-27a, ssc-miR-331-3p, ssc-miR-432-5p and ssc-miR-199a/b-3p), while 5 were down-regulated after PRV infection (ssc-mir-10a-5p, ssc-mir-148-3p, ssc-mir-219a, ssc-mir-374b-3p and ssc-miR-532-5p) (Fig 7).

**Table 4:** Fifteen miRNAs most strongly expressed in PRV-infected and uninfected samples.

Ranking	PRV-infected		Uninfected	
	miRNA	Reads	miRNA	Reads
**1**	ssc-let-7f	32,811,037	ssc-let-7f	33,388,247
**2**	ssc-let-7a	24,197,500	ssc-let-7a	23,895,506
**3**	ssc-miR-320	3,384,445	ssc-let-7d-5p	3,042,879
**4**	ssc-let-7d-5p	3,221,481	ssc-let-7e	3,002,352
**5**	ssc-let-7e	3,128,349	ssc-miR-320	2,941,979
**6**	ssc-miR-21	2,666,385	ssc-miR-21	2,452,680
**7**	ssc-miR-103	2,301,329	ssc-miR-103	2,422,357
**8**	ssc-miR-107	1,804,587	ssc-miR-140-3p	2,109,493
**9**	ssc-miR-140-3p	1,664,999	ssc-miR-107	1,956,230
**10**	ssc-let-7g	1,239,239	ssc-let-7g	1,451,004
**11**	ssc-miR-221-3p	963,000	ssc-miR-10a-5p	1,203,474
**12**	ssc-let-7c	948,125	ssc-let-7c	1,178,695
**13**	ssc-miR-423-5p	931,315	ssc-miR-185	1,017,287
**14**	ssc-miR-185	912,415	ssc-miR-423-5p	924,473
**15**	ssc-miR-29a	813,115	ssc-miR-192	873,400

## Discussion

The identification of viral and host miRNAs has had a major impact on our understanding of both the herpesvirus life cycle and of host-virus interactions [9]. Our use of solexa sequencing allowed us to find 25 additional PRV miRNAs from PRV JS-2012 infected PK15 cells. Twenty of them were confirmed through stem-loop RT-qPCR excepted prv-miR-1, 6, 16, 21 and 25 (Fig 2). In addition, using stem-loop RT-qPCR, the expression levels of 25 novel miRNAs were detected in SC or Bartha-K61infected cells. Different from the results of JS-2012, the prv-miR-3 did not detected in the SC infected cells, prv-miR-3 and 15 did not detected in the Bartha-K61 infected cells (S2 File). However, the expression levels of novel miRNAs encoded by SC or Bartha-K61 were largely consistent with the PRV variant JS-2012 strain.

Unlike the miRNAs encoded by other α-herpesviruses and γ-herpesviruses, which are found clustered in regions of the genome close to and within the latency-associated transcript (LAT) [11–13]. This study is the first to confirm the viral miRNAs encoded within open reading frames (ORFs), IRS and TRS regions of the PRV genome like β-herpesviruses [23]. We collected sRNA samples from PK15 cells infected with PRV JS-2012 at nine time points to detect comprehensively the virally encoded miRNAs, potentially explaining why we observed more miRNAs as compared with previous studies [14].

So far, PRV has been found to encode 11 miRNAs in its LLT intron region [14–16]. A recent study reported that the deletion of 9 miRNAs cluster within LAT had no effect on the ability of the virus to establish latency, speculated that a potential role for these miRNAs may be in mediating the immune response and maintaining a latent state in ganglia [24]. In our study, 6 of 11 virally encoded known miRNAs share conserved, but not completely identical sequences with miRNAs in miRBase. miRNA recognition of target mRNAs is highly dependent upon full sequence complementarity of the “seed region”, the 2–8 nts from the 5’ end of the miRNA [25]. Thus, terminal stability of the 5’ miRNA end is crucial for target discrimination. We speculate that the functions of these miRNAs may not be affected because the base changes of the 6 miRNAs are basically not located in the “seed region”. In general, the 11 known mature miRNAs sequences encoded in the LLT region were highly expressed and conserved in PRV JS-2012-infected PK15 cells.

Several studies have demonstrated that viral mRNAs can be regulated by herpesvirus miRNAs [9, 13, 26]. Herpesvirus-encoded miRNAs target crucial trans-activator proteins, such as the miR-UL112-1 targeting of the IE72 gene of HCMV [27], miR-H2-3p and miR-H6 targeting the ICP0 and ICP4 genes of HSV-1 [28], miR-H2-3 targeting the ICP0 gene of HSV-2 [29, 30] and miR-K12-7-5p targeting the RTA gene of KSHV [31]. These regulatory mecahnisms are thought to be pivotal in controlling latency establishment and reactivation, in all three herpesvirus subfamilies [26]. In our study, prv-miR-6, prv-miR-15, prv-miR-16, prv-miR-LLT-1, prv-miR-LLT-7, prv-miR-LLT-9 and prv-miR-LLT-11 are predicted to target the 3’ UTR of the PRV trans-activator IE180. IE180 is the functional equivalent of ICP0 of HSV. This may be a survival mechanism for the tight control of latency by inhibiting immediate early genes. prv-miR-5, prv-miR-15, prv-miR-16, prv-miR-17, prv-miR-22, prv-miR-23, prv-miR-25, prv-miR-LLT-1, prv-miR-LLT-3, prv-miR-LLT-7, prv-miR-LLT-8 and prv-miR-LLT-11 were predicted to target the LLT region that serves as the large latency transcript of the PRV. This targeting may be connected to maintaining a latent state or reactivation in the trigeminal ganglion.

Moreover, miRNA-regulated pathways are linked to many aspects of host-virus interaction. Viral miRNAs have a regulatory effect on cellular transcripts to exert their functions [26]. Some virus-encoded miRNAs target cellular signal pathway genes. For example, the antiproliferative and anti-angiogenic gene THBS1 is inhibited by KSHV miR-K12-6-3p [32]. The antiviral factor MICB is downregulated by HCMV miR-UL112-1, EBV miR-BART2-5p and KSHV miR-K12-7 [33]. The interferon-inducible T-cell chemoattractant CXCL11 is downregulated by EBV miR-BHRF1-3 [34]. Other virally-encoded miRNAs regulate antiviral responses and immune evasion, including EBV miR-BART-18-5p targeting of MAP3K2 [35], KSHV miR-K12-1 targeting of IkBa gene, and miR-K12-3, 7 and 11 targeting of NFIB gene [36]. KSHV miR-K12-5, 9, 10a and 10b target the BCLAF1 gene, to affect latent/lytic replication by sensitizing cells to reactivation [37]. KSHV miR-UL112-1 targets Bach-1 with pro-apoptotic activity [32]. In our study, GO analysis on the host targets of viral miRNAs revealed that these targets were involved in complex cellular pathways, including the metabolic pathway, inflammatory response, biological regulation, signaling process and immune response.

Virus can affect host miRNA expression profiles to facilitate their replication. In our study, the 13 differentially expressed host known miRNAs existed in PRV-infected sample library compared with mock sample library (S5 Table). These dysregulated host miRNAs play an important role in other viral infections. miR-132 is up-regulated in murine corneas after HSV-1 infection, facilitating viral blinding lesion effect by pathological angiogenesis [38]. miR-132 is also upregulated after KSHV infection and has a negative effect on the expression of interferon-stimulated genes through suppression of the p300, facilitating viral replication [39]. More recently, EBV and VSV have been shown both to significantly upregulate the expression of miRNA-146a and to act as a miRNA-146a-mediated negative regulator of tumor necrosis factor receptor-associated factor 6 (TRAF6), and interleukin-1 receptor-associated kinase-1 (IRAK1), key elements of the host immune and inflammatory response [40]. These findings suggest that miR-146a/b may function as novel negative regulators that help to fine-tune the immune response [41–43]. miR-199a-3p was recently reported to suppress HBV replication by targeting HBs genes [44]. Expression of miR-148 is regarded as a potential biomarker in non-small-cell lung cancer [45]. However, it has been unclear what role, if any, these dysregulated host miRNAs play in the process of PRV replication.

In conclusion, this study is the first to analyze the conservative of viral miRNAs and identified more new viral miRNAs from the PK-15 cells infected with variant PRV strain. Among 25 novel viral miRNAs, at least 17 viral miRNAs are throughout the variant PRV JS-2012 genome outside of the LLT region like β-herpesviruses. This is a breakthrough in the study of PRV miRNA, and even in α-herpesviruses. In addition, we identified the differentially expressed host miRNAs from the infected and uninfected PK 15 cells. Our findings describe the PRV virus-host interaction at the overall miRNA level. We believe these data will contribute to understand the molecular characteristics, pathogenesis and evolution trend of PRV and other herpesviruses.

## Supporting Information

S1 File The expression profiling of PRV miRNA and pre-miRNA hairpin structures. (PDF)

S2 File Expression levels detection of SC and Bartha-K61 encoded novel miRNAs using stem-loop qRT-PCR. (PDF)

S1 Table Primers used to amplify virally and differentially expressed host miRNAs by stem-loop RT-qPCR. (DOCX)

S2 Table Predicted host target genes of viral miRNAs. (XLSX)

S3 Table GO term enrichment analysis of swine target genes. Total predicted host targets were performed by GO enrichment analysis using GO::TermFinder (P < 0.05). (XLSX)

S4 Table Novel porcine miRNA expressed in PK15 cells. (DOCX)

S5 Table Differentially expressed host known miRNA in PRV-infected PK15 cells. (XLSX)
