# Supplementary material for: Identification and Analysis of Novel Viral and Host Dysregulated MicroRNAs in Variant Pseudorabies Virus-Infected PK15 Cells
Source: PLoS One. 2016 Mar 21;11(3):e0151546. doi: 10.1371/journal.pone.0151546 (PMC4801506; doi:10.1371/journal.pone.0151546)

S1 File. The expression profiling of PRV miRNA and pre-miRNA hairpin structures.

miRNA sequences and their coresponding reads mapped on the precursors of 25 PRV novel miRNA genes. The sequences of the miRNA genes and their secondary structures (shown in dot-bracket notation) predicted by Miread soft. Mature RNAs are shown in red and relevant miRNA* are shown in green. The hairpin structures of pre-miRNA is shown at the back.

prv-miR-1

```
CTCTCCGACAGGCGGTCGGGGGGCGCGTCGGGCCCGCTGTCGGGACGATCCGCCTCCGGCCGCCGCCGCTGGTGCT 479
..(.(((...((((((((((((.((.((((..((((....)))).)))).))))))))))))))).....))).).. -41.80(kcal/mol)
**********GGCGGTCGGGGGGCGCGTCGGGC******************************************* prv-miR-1-5p 479
-----CGACAGGCGGTCGGGGGGCGCGTCGG--------------------------------------------- 1
-------ACAGGCGGTCGGGGGGCGCG------------------------------------------------- 1
-------ACAGGCGGTCGGGGGGCGCGTCGGGC------------------------------------------- 2
--------CAGGCGGTCGGGGGGCGCGTCG---------------------------------------------- 1
--------CAGGCGGTCGGGGGGCGCGTCGG--------------------------------------------- 1
--------CAGGCGGTCGGGGGGCGCGTCGGG-------------------------------------------- 3
--------CAGGCGGTCGGGGGGCGCGTCGGGC------------------------------------------- 10
---------AGGCGGTCGGGGGGCGCGT------------------------------------------------ 3
---------AGGCGGTCGGGGGGCGCGTCGGG-------------------------------------------- 2
---------AGGCGGTCGGGGGGCGCGTCGGGC------------------------------------------- 4
----------GGCGGTCGGGGGGCGCGTCGG--------------------------------------------- 1
----------GGCGGTCGGGGGGCGCGTCGGG-------------------------------------------- 88
----------GGCGGTCGGGGGGCGCGTCGGGC------------------------------------------- 314
----------GGCGGTCGGGGGGCGCGTCGGGCC------------------------------------------ 30
----------GGCGGTCGGGGGGCGCGTCGGGCCC----------------------------------------- 2
-----------GCGGTCGGGGGGCGCGTCGGGC------------------------------------------- 2
------------CGGTCGGGGGGCGCGTCGG--------------------------------------------- 1
------------CGGTCGGGGGGCGCGTCGGG-------------------------------------------- 5
------------CGGTCGGGGGGCGCGTCGGGC------------------------------------------- 2
-------------GGTCGGGGGGCGCGTCGGGC------------------------------------------- 4
--------------GTCGGGGGGCGCGTCGGGC------------------------------------------- 2
```

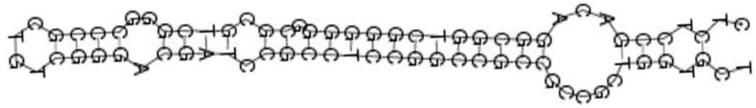

prv-miR-2

```
AACGCGCGGCGGCTCGCGGACACGTGCCTGGCGGCGCTCATGCACCTGTACCTCTCGGTCGGCGCCGT 139
.(((.(((.((((.((.(((((.((((.((((...)).)).)))).))).))...)))))).)))))) -29.10(kcal/mol)
**************CGCGGACACGTGCCTGGC************************************ prv-miR-2-5p 1
**************************************CATGCACCTGTACCTCTCGG********** prv-miR-2-3p 136
----------------CGGACACGTGCCTGGCGGCGCT------------------------------  1
-----------------------GTGCCTGGCGGCGCTCATGCAC-----------------------  1
-------------------------GCCTGGCGGCGCTCATGCAC-----------------------  1
-----------------------------------GCTCATGCACCTGTACCTCTCGG----------  3
------------------------------------CTCATGCACCTGTACCTCTCGG----------  4
-------------------------------------TCATGCACCTGTACCTCTCGG----------  3
--------------------------------------CATGCACCTGTACCTCTCGG----------  73
--------------------------------------CATGCACCTGTACCTCTCGGTCGGC-----  2
---------------------------------------ATGCACCTGTACCTCTCGG----------  4
----------------------------------------TGCACCTGTACCTCTCGG----------  47
```

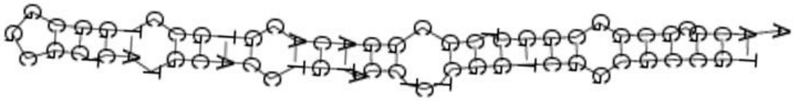

prv-miR-3

```
CGAGGCCCTGTTGGGGGGCGCGGCCCCGGGCCGGCCGCCCGTGGTGGCCGTCCCGCGGGCGGCCAGCCCGGACGCGCTGTACCGGCGCCTC 5
.((((...(((((..((((((...((((((.((((((((((((.......)))))))))))).)))))).))))))...))))))))) -68.70(kcal/mol)
***********************************************************CGGCCAGCCCGGACGCGCTGTA********** prv-miR-3-3p 5
-----------------------------------------------------------CGGCCAGCCCGGACGCGCTGTA---------- 5
```

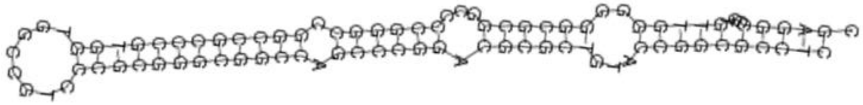

prv-miR-4

```
CGTCGCGCTACCTGAGCTCGGGGCGCCGCTGGCGCGACGACTGGGGGCGCGCGCCGCGCTTCGAC 16
((.(((((..(((.((.(((..(((((...)))))..))))).))).)))))).)).........  -33.70(kcal/mol)
**********************************CGACGACTGGGGGCGCGCGCC********** prv-miR-4-3p 16
```

--------------------------------CGCGACGACTGGGGGCGCGCGC----------- 2
---------------------------------GCGACGACTGGGGGCGCGCGCC---------- 1
----------------------------------CGACGACTGGGGGCGCGC------------- 1
----------------------------------CGACGACTGGGGGCGCGCGC----------- 2
----------------------------------CGACGACTGGGGGCGCGCGCC---------- 5
-----------------------------------GACGACTGGGGGCGCGCGC----------- 1
------------------------------------ACGACTGGGGGCGCGCGCCG--------- 1
------------------------------------ACGACTGGGGGCGCGCGCCGC-------- 2
-------------------------------------CGACTGGGGGCGCGCGCCGCG------- 1

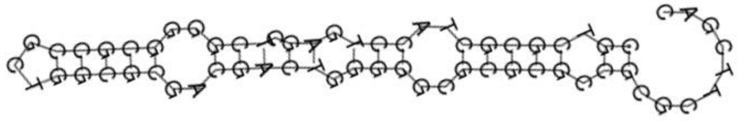

prv-miR-5

GAGCTCTGCCGCCCGCGCGACGACCCCGAGCTCCAGGACACCCTCTTCGGCGCGTACAAGGCGCCCGAGCTCTGCGACCGGCGCGGGCGCCCGCT 14
.(((...(.(((((((((..((..((.((((((.(((....)))....(((((.......))))).)))))).).)..)))))))))))).).))) -41.70(kcal/mol)
CGAGCTCTGCGACCGGCGCG******* prv-miR-5-3p 10
--------------------------------CCAGGACACCCTCTTCGGCG--- 2
----------------------------------AGGACACCCTCTTCGGCGC-- 1
---GCGTACAAGGCGCCCGAGCTCT---------------------- 1
---CGAGCTCTGCGACCGGCGCG---------- 10

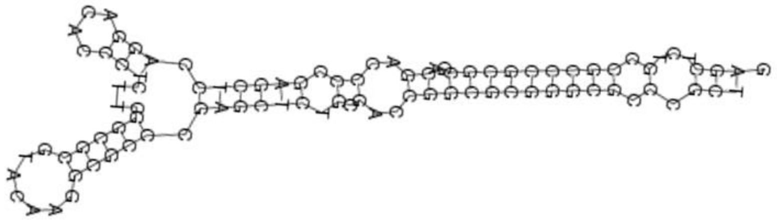

prv-miR-6

GCCGTGGCGGCGCAGGCGCGCGGCATGGAGGTGACGGAGTCCGCGTACGGCGACCACATCCGGCAGTGCGTGTGCGCCTTCACGTCG 11
..(((((.((((((.((((((.((.((((.(((..(..(.(((....))))..)))).)))))).)))))).)))))).)))))... -46.60(kcal/mol)
**********CGCAGGCGCGCGGCATGGAGGT*** prv-miR-6-5p 10
----------CGCAGGCGCGCGGCATGGAGGT--- 6

-----------GCAGGCGCGCGGCATGGAGGT---   4
-----------------GCGCGGCATGGAGGTGACGG--   1

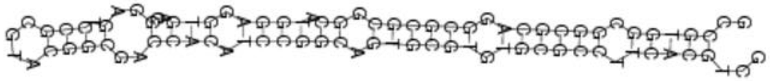

prv-miR-7

GGCCTTCTTCTACCCGGCGCCCGTGAACGCGCTCTTTGCCTGCGCCGACCACCTCGGCGCCATGCGCGACGTGGGCGCCGAGGTGCGCGCCGCCG 8
(((.......(((((((((((((((..(((((.........(((((((.....)))))))...))))).))))))))))).))))......))). -48.90(kcal/mol)
**********TACCCGGCGCCCGTGAACGCGCTC*** prv-miR-7-5p 5
CGCGACGTGGGCGCCGAGGTG******** prv-miR-7-3p 2
----------TACCCGGCGCCCGTGAACGCGCTC--- 4
----------TACCCGGCGCCCGTGAACGCGCTCT-- 1
--CGCGACGTGGGCGCCGAGGTG---------- 2
--GTGGGCGCCGAGGTGCGCGCCGCCG 1

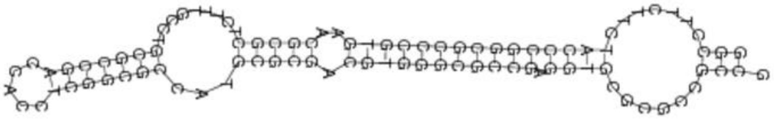

prv-miR-8

GGCGGTGGCGCTGCGCGTGGGCGCGCGCGAGCTGATCTTCACCTACGACGAGCTCCTGCCGGCCCGCACGCGCTACAACG 10
.(..(((((((.(.(((.((.((.(((.(((((..((.........))..))))).))))).))))).))))))))..). -38.40(kcal/mol)
CGAGCTCCTGCCGGCCCGCACG******** prv-miR-8-3p 9
-------------------------CGCGAGCTGATCTTCACCTACG--------------------------------- 1
--GACGAGCTCCTGCCGGCCCGCACGCG-------- 2
--CGAGCTCCTGCCGGCCCGCAC----------- 1
--CGAGCTCCTGCCGGCCCGCACG---------- 6

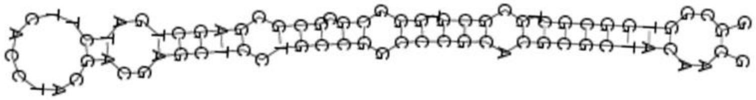

prv-miR-9

GGCCGGCGCGTACGCGGCGCGCTTCGTCCACGTGACGGCGGAGCCGTGGACGGGCTCGGCGGCCGCGGTGGCCGCGGCGCTGCGCGACGCGAGCGGCA 47
.((((.(((((.((((..(((((.((.(((((((.(.((.(((((......))))).)).).))).)))).)).)))))..)))))))))..)))). -62.80(kcal/mol)
**********TACGCGGCGCGCTTCGTCCACG** prv-miR-9-5p 6
----------TACGCGGCGCGCTTCGTCCACG-- 6
--------------------------------TGACGGCGGAGCCGTGGACGGGCT-- 1
-----------------------------------CGGCGGAGCCGTGGACGGGCT-- 1
------------------------------------GGCGGAGCCGTGGACGGG-- 2
--------------------------------------CGGAGCCGTGGACGGGCTCGGCG------------------------------------- 1
---AGCCGTGGACGGGCTCGGCGGCC---------------------------------- 5
---CCGTGGACGGGCTCGGCGGCCGC-------------------------------- 1
--CGTGGACGGGCTCGGCGGC----------------------------------- 3
--CGTGGACGGGCTCGGCGGCCGC-------------------------------- 4
--CGTGGACGGGCTCGGCGGCCGCG------------------------------- 5
---GTGGACGGGCTCGGCGGCCGC-------------------------------- 1
---GTGGACGGGCTCGGCGGCCGCG------------------------------- 1
--TGGACGGGCTCGGCGGCCGCG------------------------------- 14
--TGGACGGGCTCGGCGGCCGCGGTG---------------------------- 1
--GACGGGCTCGGCGGCCGCGGTG---------------------------- 1

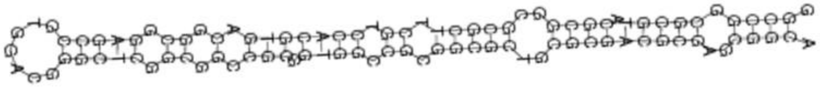

prv-miR-10

CGGCGGCGTGCCCGCGGACGCGCCGGAGGCGGCCGCCGCCGCGGCCGAGCGCGTGCGCGCCGCCT 105
.((((((((((.((((..((.((((..((((((....)))))).))))))..)))).)))))))))). -54.90(kcal/mol)
**********CCCGCGGACGCGCCGGAGGCGG********************************* 10-5p 105
----------CCCGCGGACGCGCCGGAGGCG---------------------------------- 5
----------CCCGCGGACGCGCCGGAGGCGG--------------------------------- 86
----------CCCGCGGACGCGCCGGAGGCGGC-------------------------------- 8
-----------CCGCGGACGCGCCGGAGGCG---------------------------------- 1
-----------CCGCGGACGCGCCGGAGGCGG--------------------------------- 3
-----------CCGCGGACGCGCCGGAGGCGGC-------------------------------- 2

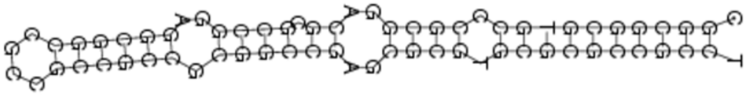

prv-miR-11

```
CTGTCCCTCATCCACTTCTCGACGGCCGGCTCCCCGCGGGGCGGGGGCTGCGGGGGGGTGGGTGGGTGATAGA 29
(((((.((((((((((((((..(((((.(((((....)))))...)))))..)))))))))))))).))))). -46.00(kcal/mol)
**********TCCACTTCTCGACGGCCGGCTCC**************************************** prv-miR-11-5p 28
----------TCCACTTCTCGACGGCCGGCTCC----------------------------------------  27
------------CACTTCTCGACGGCCGGCTCCC---------------------------------------  1
-----------------------------------------------------GGGGGTGGGTGGGTGATA--  1
```

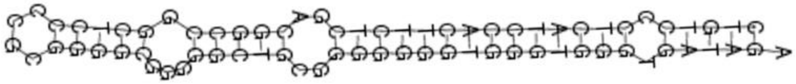

prv-miR-12

```
CATTGGCTCCCCTCGCGCCACGCGGTCGCCGTCGTCATCGTCCCGCCGGCCAATCGGGTGGCAGCGGGGGAGGCTGGGAG 414
..((((((.((((((((((((.((((.((((.((.........)).))))..)))).))))).))))))).))))))... -42.90(kcal/mol)
**********CCTCGCGCCACGCGGTCGCCGTCG********************************************** prv-miR-12-5p 6
*************************************************CCAATCGGGTGGCAGCGGGGG********** prv-miR-12-3p 400
----------CCTCGCGCCACGCGGTCGCCGTCG----------------------------------------------  2
----------CCTCGCGCCACGCGGTCGCCGTCGT---------------------------------------------  2
------------TCGCGCCACGCGGTCGCCGTCGTC--------------------------------------------  1
--------------GCGCCACGCGGTCGCCGT------------------------------------------------  1
---------------CGCCACGCGGTCGCCGTCGTCATC-----------------------------------------  1
--------------------CGCGGTCGCCGTCGTCATCGTCCC------------------------------------  1
-------------------------------TCGTCATCGTCCCGCCGGCC-----------------------------  1
--------------------------------CGTCATCGTCCCGCCGGCC-----------------------------  1
---------------------------------GTCATCGTCCCGCCGGCCA----------------------------  1
---------------------------------GTCATCGTCCCGCCGGCCAATC-------------------------  2
--------------------------------------------GCCGGCCAATCGGGTGGCAGCGGGGG----------  1
----------------------------------------------CGGCCAATCGGGTGGCAGCGGGGG----------  1
------------------------------------------------GCCAATCGGGTGGCAGCGGGGG----------  10
------------------------------------------------GCCAATCGGGTGGCAGCGGGGGA---------  2
------------------------------------------------GCCAATCGGGTGGCAGCGGGGGAGG-------  2
-------------------------------------------------CCAATCGGGTGGCAGCGGG------------  10
```

```
-------------------------------------------------CCAATCGGGTGGCAGCGGGG-----------  2
-------------------------------------------------CCAATCGGGTGGCAGCGGGGG----------  235
-------------------------------------------------CCAATCGGGTGGCAGCGGGGGA---------  2
--------------------------------------------------CAATCGGGTGGCAGCGGG------------  7
--------------------------------------------------CAATCGGGTGGCAGCGGGG-----------  1
--------------------------------------------------CAATCGGGTGGCAGCGGGGG----------  89
--------------------------------------------------CAATCGGGTGGCAGCGGGGGA---------  2
--------------------------------------------------CAATCGGGTGGCAGCGGGGGAGGC------  7
---------------------------------------------------AATCGGGTGGCAGCGGGGG----------  9
---------------------------------------------------AATCGGGTGGCAGCGGGGGA---------  1
---------------------------------------------------AATCGGGTGGCAGCGGGGGAGGC------  2
----------------------------------------------------ATCGGGTGGCAGCGGGGG----------  3
----------------------------------------------------ATCGGGTGGCAGCGGGGGAG--------  1
----------------------------------------------------ATCGGGTGGCAGCGGGGGAGGC------  2
-----------------------------------------------------TCGGGTGGCAGCGGGGGAG--------  10
-----------------------------------------------------TCGGGTGGCAGCGGGGGAGGCTGG---  1
-----------------------------------------------------TCGGGTGGCAGCGGGGGAGGCTGGGA-  1
```

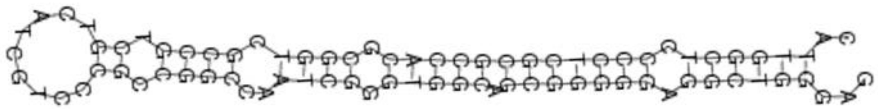

prv-miR-13

```
CGCGCGCGCCCCGGGGAAGGGTCGGGCGATGGCCGCCGCCACCGCCCCACCCTTCCCCCGGACCCCCTCCCGCC  1000
.(((.(.(.((.((((((((((.(((((.((((....)))).))))).)))))))))).)).....).).))).  -42.80(kcal/mol)
**********CCGGGGAAGGGTCGGGCGATG*******************************************  prv-miR-13-5p 983
*****************************************CCGCCCCACCCTTCCCCCGGACC**********  prv-miR-13-3p 9
--CGCGCGCCCCGGGGAAGGGTCGG-------------------------------------------------  1
--CGCGCGCCCCGGGGAAGGGTCGGG------------------------------------------------  2
---GCGCGCCCCGGGGAAGGGTCGGG------------------------------------------------  3
--------CCCCGGGGAAGGGTCGGG------------------------------------------------  1
--------CCCCGGGGAAGGGTCGGGC-----------------------------------------------  2
--------CCCCGGGGAAGGGTCGGGCG----------------------------------------------  2
--------CCCCGGGGAAGGGTCGGGCGA---------------------------------------------  6
```

```
--------CCCCGGGGAAGGGTCGGGCGAT--------------------------------------------   47
--------CCCCGGGGAAGGGTCGGGCGATG-------------------------------------------   68
--------CCCCGGGGAAGGGTCGGGCGATGG------------------------------------------   14
--------CCCCGGGGAAGGGTCGGGCGATGGC-----------------------------------------   36
--------CCCCGGGGAAGGGTCGGGCGATGGCC----------------------------------------   6
---------CCCGGGGAAGGGTCGGGC-----------------------------------------------   1
---------CCCGGGGAAGGGTCGGGCG----------------------------------------------   2
---------CCCGGGGAAGGGTCGGGCGA---------------------------------------------   1
---------CCCGGGGAAGGGTCGGGCGAT--------------------------------------------   19
---------CCCGGGGAAGGGTCGGGCGATG-------------------------------------------   7
---------CCCGGGGAAGGGTCGGGCGATGG------------------------------------------   2
---------CCCGGGGAAGGGTCGGGCGATGGC-----------------------------------------   15
---------CCCGGGGAAGGGTCGGGCGATGGCC----------------------------------------   14
----------CCGGGGAAGGGTCGGGCGA---------------------------------------------   4
----------CCGGGGAAGGGTCGGGCGAT--------------------------------------------   22
----------CCGGGGAAGGGTCGGGCGATG-------------------------------------------   309
----------CCGGGGAAGGGTCGGGCGATGG------------------------------------------   42
----------CCGGGGAAGGGTCGGGCGATGGC-----------------------------------------   54
----------CCGGGGAAGGGTCGGGCGATGGCC----------------------------------------   35
-----------CGGGGAAGGGTCGGGCGAT--------------------------------------------   2
-----------CGGGGAAGGGTCGGGCGATG-------------------------------------------   107
-----------CGGGGAAGGGTCGGGCGATGG------------------------------------------   22
-----------CGGGGAAGGGTCGGGCGATGGC-----------------------------------------   110
-----------CGGGGAAGGGTCGGGCGATGGCC----------------------------------------   12
------------GGGGAAGGGTCGGGCGATG-------------------------------------------   1
------------GGGGAAGGGTCGGGCGATGG------------------------------------------   5
------------GGGGAAGGGTCGGGCGATGGC-----------------------------------------   6
-------------GGGAAGGGTCGGGCGATG-------------------------------------------   1
-------------GGGAAGGGTCGGGCGATGG------------------------------------------   3
-------------GGGAAGGGTCGGGCGATGGC-----------------------------------------   2
--------------GGAAGGGTCGGGCGATGGCCGC--------------------------------------   3
---------------GAAGGGTCGGGCGATGGCCGCCGCCA---------------------------------   2
-----------------------------------------CCGCCCCACCCTTCCCCCGGACC----------   9
```

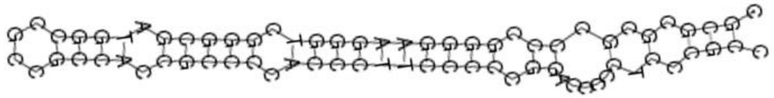

prv-miR-14

```
CGACACGCGGCGCCTCGGGGCCGACGTGAACCGCCTCTTTCGCGGCATCGCCGTCTCGGCCGCCGACGTGAC 50
...((((((((.(.((((((....((((.((((......))))..)))).))))))).))))).)))).. -32.00(kcal/mol)
**********CGCCTCGGGGCCGACGTGAACC**************************************** prv-miR-14-5p 39
*****************************************GCGGCATCGCCGTCTCGGCCG********** prv-miR-14-3p 10
----------CGCCTCGGGGCCGACGTGAA------------------------------------------ 1
----------CGCCTCGGGGCCGACGTGAACC---------------------------------------- 33
------------CCTCGGGGCCGACGTGAAC----------------------------------------- 1
------------CCTCGGGGCCGACGTGAACCGC-------------------------------------- 3
-------------CTCGGGGCCGACGTGAACC---------------------------------------- 1
------------------GGCCGACGTGAACCGCCTCTT--------------------------------- 1
-----------------------------------------GCGGCATCGCCGTCTCGGCCG---------- 8
---------------------------------------------CATCGCCGTCTCGGCCGCCGACG---- 2
```

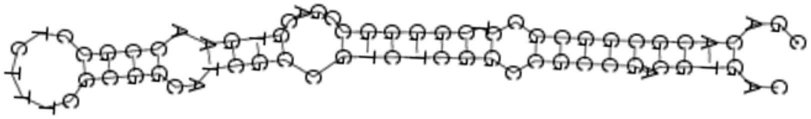

prv-miR-15

```
GCCCCGAGCCTCTCCGCCGAGACGACCCCGGGCCCCGTCACCGAGGTCCCGAGTCCTCGGCCGAGGTCTGGGAC 7
..((((.(((((...((((((..(((.(.(((((.((....)).))))).).))).)))))).))))).)))).. -38.30(kcal/mol)
**********TCTCCGCCGAGACGACCCCGGGC***************************************** prv-miR-15-5p 7
----------TCTCCGCCGAGACGACCCCGG------------------------------------------- 2
----------TCTCCGCCGAGACGACCCCGGGC----------------------------------------- 3
----------TCTCCGCCGAGACGACCCCGGGCC---------------------------------------- 1
------------TCCGCCGAGACGACCCCGGGCC---------------------------------------- 1
```

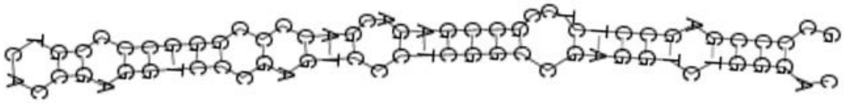

prv-miR-16

```
GGCGCGCTCCGCGCTCCCCCGCCGCCTGGGCGGGGATGCCGCGCCGGCACGAGCGCACGCGGGCGCGCGGGCGGCGGGAGGGGAGGGAGGAGGAAGGG 96
......((((.(.((((((((((((((.(((....(((.((((((...)).))))..)))..))).))))))))))...)))))).).))))...... -58.90(kcal/mol)
*****************************************************************CGCGGGCGGCGGGAGGGGAGGGA********** prv-miR-16-3p 96
---------------------------------------------------------------CGCGCGGGCGGCGGGAGGGGAGGG----------- 1
---------------------------------------------------------------CGCGCGGGCGGCGGGAGGGGAGGGA---------- 2
----------------------------------------------------------------GCGCGGGCGGCGGGAGGGGAGGGA---------- 1
-----------------------------------------------------------------CGCGGGCGGCGGGAGGGGAGGGA---------- 86
------------------------------------------------------------------GCGGGCGGCGGGAGGGGAGGG----------- 1
-------------------------------------------------------------------CGGGCGGCGGGAGGGGAGGGA---------- 5
```

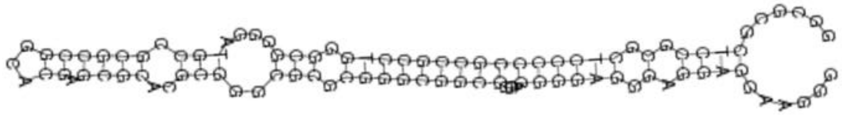

prv-miR-17

```
GACCCCCGGTGCGACGGAAGGGGCAGGCTCGGCGCGCCGATGCGATCCGCGGAGCCCGCCCCGTCCGCGCCCCGGAAGCCGG 5690
.....((((.(((.((((.(((((.(((((.((((((....)))...))).))))).))))).))))))).)))))....... -50.70(kcal/mol)
**********GCGACGGAAGGGGCAGGCTCGGCG************************************************ prv-miR-17-5p 5635
*************************************************CGGAGCCCGCCCCGTCCGCGCCC********** prv-miR-17-3p 37
----CCCGGTGCGACGGAAGGGGCAGG------------------------------------------------------- 1
----CCCGGTGCGACGGAAGGGGCAGGCT----------------------------------------------------- 1
------CGGTGCGACGGAAGGGGCAGG------------------------------------------------------- 1
------CGGTGCGACGGAAGGGGCAGGCTC---------------------------------------------------- 5
-------GGTGCGACGGAAGGGGCAGG------------------------------------------------------- 2
--------GTGCGACGGAAGGGGCAGGCTCGG-------------------------------------------------- 2
---------TGCGACGGAAGGGGCAGGCT----------------------------------------------------- 2
---------TGCGACGGAAGGGGCAGGCTC---------------------------------------------------- 1
---------TGCGACGGAAGGGGCAGGCTCG--------------------------------------------------- 12
---------TGCGACGGAAGGGGCAGGCTCGG-------------------------------------------------- 36
---------TGCGACGGAAGGGGCAGGCTCGGC------------------------------------------------- 1
---------TGCGACGGAAGGGGCAGGCTCGGCG------------------------------------------------ 18
----------GCGACGGAAGGGGCAGGCT----------------------------------------------------- 2
```

----------GCGACGGAAGGGGCAGGCTC--	3
----------GCGACGGAAGGGGCAGGCTCG---	57
----------GCGACGGAAGGGGCAGGCTCGG--	281
----------GCGACGGAAGGGGCAGGCTCGGC---	291
----------GCGACGGAAGGGGCAGGCTCGGCG--	2190
----------GCGACGGAAGGGGCAGGCTCGGCGC---	7
----------GCGACGGAAGGGGCAGGCTCGGCGCG--	7
-----------CGACGGAAGGGGCAGGCTC--	3
-----------CGACGGAAGGGGCAGGCTCGG--	10
-----------CGACGGAAGGGGCAGGCTCGGC---	31
-----------CGACGGAAGGGGCAGGCTCGGCG--	1148
-----------CGACGGAAGGGGCAGGCTCGGCGC---	2
-----------CGACGGAAGGGGCAGGCTCGGCGCG--	10
------------GACGGAAGGGGCAGGCTCG---	3
------------GACGGAAGGGGCAGGCTCGG--	29
------------GACGGAAGGGGCAGGCTCGGC---	33
------------GACGGAAGGGGCAGGCTCGGCG--	1083
------------GACGGAAGGGGCAGGCTCGGCGC---	2
------------GACGGAAGGGGCAGGCTCGGCGCG--	2
------------GACGGAAGGGGCAGGCTCGGCGCGCC--	2
-------------ACGGAAGGGGCAGGCTCGG--	5
-------------ACGGAAGGGGCAGGCTCGGC---	10
-------------ACGGAAGGGGCAGGCTCGGCG--	164
-------------ACGGAAGGGGCAGGCTCGGCGC---	1
-------------ACGGAAGGGGCAGGCTCGGCGCG--	4
-------------ACGGAAGGGGCAGGCTCGGCGCGC---	3
-------------ACGGAAGGGGCAGGCTCGGCGCGCCG---	1
--------------CGGAAGGGGCAGGCTCGG--	4
--------------CGGAAGGGGCAGGCTCGGC---	14
--------------CGGAAGGGGCAGGCTCGGCG--	113
--------------CGGAAGGGGCAGGCTCGGCGC---	2
--------------CGGAAGGGGCAGGCTCGGCGCG--	37
--------------CGGAAGGGGCAGGCTCGGCGCGC---	1

---------------GGAAGGGGCAGGCTCGGC--- 2
---------------GGAAGGGGCAGGCTCGGCG-- 4
---------------GGAAGGGGCAGGCTCGGCGCGC--- 1
----------------GAAGGGGCAGGCTCGGCG-- 4
----------------GAAGGGGCAGGCTCGGCGCG-- 2
----------------GAAGGGGCAGGCTCGGCGCGC--- 1
-----------------AAGGGGCAGGCTCGGCGCG-- 2
---CGGAGCCCGCCCCGTCCGCGCC----------- 6
---CGGAGCCCGCCCCGTCCGCGCCC---------- 20
--GGAGCCCGCCCCGTCCGCGCCC---------- 7
---GAGCCCGCCCCGTCCGCGC------------ 1
---GAGCCCGCCCCGTCCGCGCCC---------- 3

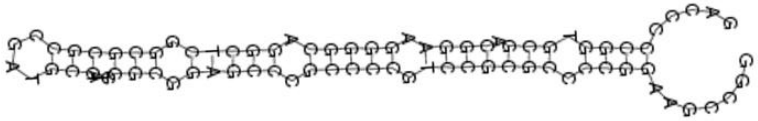

prv-miR-18

GTCCGCGTCCTCCTCCACCGCCTCGACTCCGTCCCGAGAAGCCGCCTCGTCCTCCTCTTCTTCCCCGGCCGGAACACCGAGCGATGGCTCCCGCGGA 9
.((((((......(((.(((.(((..((((.((.(.((((...................)))).).)).))))....)))))).)))....)))))) -27.50(kcal/mol)
GGCCGGAACACCGAGCGATGG******** prv-miR-18-3p 6
---CCGCCTCGTCCTCCTCTTCTT----------------------------------- 3
---CGGCCGGAACACCGAGCGATGGCTC------- 1
--GGCCGGAACACCGAGCGATGG---------- 3
--GGCCGGAACACCGAGCGATGGCT-------- 2

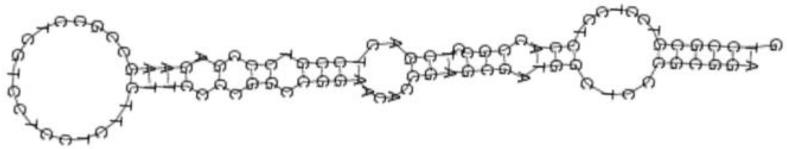

prv-miR-19

CGCCGCGGTCACGGAGCGCCTGGACGCCGCGGCGCACGTGCGCGAGGCCGCGGCGCTCGGCGCGCCGGCGCCCGGCGA 104

(((((.(((..(((.(((((.((.(((((((((.(.((....)).))))))))))))).))))).)))..)))))))).-57.10(kcal/mol)
**********ACGGAGCGCCTGGACGCCGCGGC*** prv-miR-19-5p 103
CGCCGCGGTCACGGAGCGCCTGGAC--- 1
--------TCACGGAGCGCCTGGACGCCGCGGC--- 1
---------CACGGAGCGCCTGGACGCCGCGG-- 1
---------CACGGAGCGCCTGGACGCCGCGGC--- 8
----------ACGGAGCGCCTGGACGCCGCGG-- 5
----------ACGGAGCGCCTGGACGCCGCGGC--- 57
-----------CGGAGCGCCTGGACGCCGCGGC--- 27
-----------CGGAGCGCCTGGACGCCGCGGCG-- 2
------------GGAGCGCCTGGACGCCGCGGC--- 2

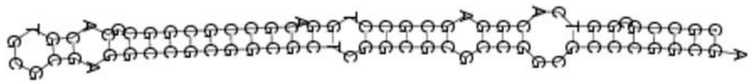

prv-miR-20

GCCCATCAACATCGACCACCGCCCGCGCTGCGACATCGGCGCCGTGCTGGCCGTGGTCGACGACGACCGCGGC 132
((((.((..(.((((((((.(((.((((.(((.(....)))).)))).))).)))))))).)..))..).)))-34.50(kcal/mol)
**********ATCGACCACCGCCCGCGCTGCGA** prv-miR-20-5p 17
CCGTGCTGGCCGTGGTCGACGA******** prv-miR-20-3p 114
--------ACATCGACCACCGCCCGCGCT-- 1
---------CATCGACCACCGCCCGCGCTG--- 1
---------CATCGACCACCGCCCGCGCTGC-- 3
---------CATCGACCACCGCCCGCGCTGCGA-- 1
----------ATCGACCACCGCCCGCGCTGC-- 3
----------ATCGACCACCGCCCGCGCTGCG--- 3
----------ATCGACCACCGCCCGCGCTGCGA-- 3
------------CGACCACCGCCCGCGCTGCGAC--------------------------------------- 2
---CCGTGCTGGCCGTGGTCGACG----------- 8
---CCGTGCTGGCCGTGGTCGACGA---------- 68
---CCGTGCTGGCCGTGGTCGACGAC--------- 2
--CGTGCTGGCCGTGGTCGACG----------- 1

```
------------------------------------------CGTGCTGGCCGTGGTCGACGA----------   21
------------------------------------------CGTGCTGGCCGTGGTCGACGAC---------   6
------------------------------------------CGTGCTGGCCGTGGTCGACGACG--------   1
--------------------------------------------TGCTGGCCGTGGTCGACGACGA-------   7
----------------------------------------------CTGGCCGTGGTCGACGACGA-------   1
```

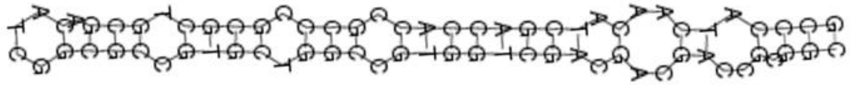

prv-miR-21

```
GCGCCGCGCAGGCGCACGCAGCGCCGCGAGCACCGCGCGCCGCCGCGCCCGGCGGTCGCCCCGGCGCACGCGGCGGGGGCGAGGGCGTCCCCCGGGGCGG 8
.((((.((..(((((.(((..((((((.((.(((.((((((((....)))))).))).)))..))).)).)))))))...)))...))).))..)).)))). -64.00(kcal/mol)
******************************************************************CACGCGGCGGGGGCGAGGGCGTCC********** prv-miR-21-3p 7
----------------------------AGCACCGCGCGCCGCCGCGCCC--------------------------------------------------   1
------------------------------------------------------------------CACGCGGCGGGGGCGAGGGCGTCC----------   7
```

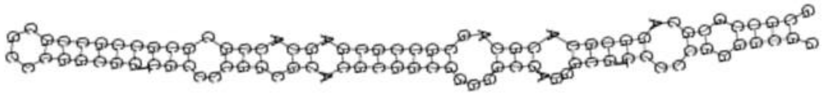

prv-miR-22

```
CCGCCGGGTTCGGGCCCCTGCTCGGCCTGCCGGGCGACGCGCCGCCGCCCGACGGGCTGGTGCGGACGCGCCCCGGCG 105
.((((((...(.((..(((((((((((.((((((.((...)).)))))).)))))))).))))..)).)))))))) -50.10(kcal/mol)
***********************************************CCCGACGGGCTGGTGCGGACG********** prv-miR-22-3p 105
----------------------------------------------GCCCGACGGGCTGGTGCGGA------------   1
-----------------------------------------------CCCGACGGGCTGGTGCGGAC-----------   1
-----------------------------------------------CCCGACGGGCTGGTGCGGACG----------   72
-----------------------------------------------CCCGACGGGCTGGTGCGGACGC---------   17
-----------------------------------------------CCCGACGGGCTGGTGCGGACGCG--------   4
-----------------------------------------------CCCGACGGGCTGGTGCGGACGCGC-------   7
------------------------------------------------CCGACGGGCTGGTGCGGACGCGC-------   2
--------------------------------------------------GACGGGCTGGTGCGGACGCG--------   1
```

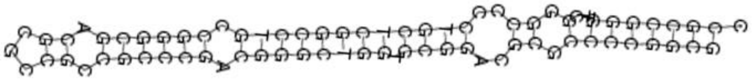

prv-miR-23

```
AGTACATCGACTGGATCGTGTGCCTCGGCGTGGTCCCGGTGCGCCGGGCGCGCCCCGGGGGGAAGCGG 11
......((..((((...(((((((.(((((((.......)))))))))))))).))))..))...... -30.20(kcal/mol)
**********CTGGATCGTGTGCCTCGGCGTG************************************ prv-miR-23-5p 11
-------CGACTGGATCGTGTGCCTCGGC---------------------------------------  1
---------ACTGGATCGTGTGCCTCGGCG--------------------------------------  2
----------CTGGATCGTGTGCCTCGGCGTG------------------------------------  4
----------CTGGATCGTGTGCCTCGGCGTGGT----------------------------------  2
-----------TGGATCGTGTGCCTCGGCGTG------------------------------------  2
```

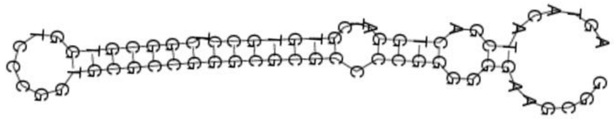

prv-miR-24

```
GGGCGGCGACGCGCGTCGAGCCCGTGATGACGCAGTCCATGGTCTCGTTGAGGGTCTGGATGCACGTGCTCTTGCC 36
.((((((.(((.(((((.(((((.((((((.((........)).)))))).))).)).))))).))))))...))) -33.20(kcal/mol)
***********CGCGTCGAGCCCGTGATGAC********************************************* prv-miR-24-5p 1
*********************************************CGTTGAGGGTCTGGATGCACG********** prv-miR-24-3p 34
-------GACGCGCGTCGAGCCCGTGAT------------------------------------------------  1
--------------------------------------ATGGTCTCGTTGAGGGTCTGGA----------------  1
-------------------------------------------CTCGTTGAGGGTCTGGATGCA------------  1
--------------------------------------------TCGTTGAGGGTCTGGATGCAC-----------  1
---------------------------------------------CGTTGAGGGTCTGGATGC-------------  1
---------------------------------------------CGTTGAGGGTCTGGATGCA------------  2
---------------------------------------------CGTTGAGGGTCTGGATGCACG----------  14
---------------------------------------------CGTTGAGGGTCTGGATGCACGT---------  2
---------------------------------------------CGTTGAGGGTCTGGATGCACGTGC-------  10
----------------------------------------------GTTGAGGGTCTGGATGCACG----------  1
-----------------------------------------------TTGAGGGTCTGGATGCAC-----------  1
-----------------------------------------------TTGAGGGTCTGGATGCACGTGCT------  1
```

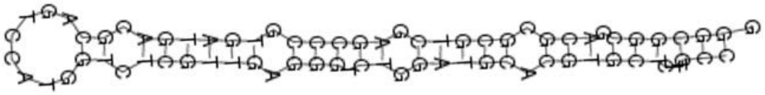

prv-miR-25

```
TACGGGTTCCACGGGCGCGCGGGCGGCGCCGGCGTCGTGACGCCGTCCACGTAGATCGCGTCGTCGCCGCCGCCGGGGCCCCACGGGGTGCCCGGG 28
..(((((.((.((((.((.(.(((((((.(((((.((((((..((....))..).))))).))))).))))))).).)).)).)).)))))).. -58.10(kcal/mol)
**********ACGGGCGCGCGGGCGGCGCCGG**************************************************************** prv-miR-25-5p 5
----------ACGGGCGCGCGGGCGGCGCCGG---------------------------------------------------------------- 5
----------------------------------------CGCCGTCCACGTAGATCGCGTCGT-------------------------------- 2
----------------------------------------CGCCGTCCACGTAGATCGCGTCGTC------------------------------- 1
-----------------------------------------GCCGTCCACGTAGATCGCGTCG--------------------------------- 1
------------------------------------------CCGTCCACGTAGATCGCGTCGT-------------------------------- 3
------------------------------------------CCGTCCACGTAGATCGCGTCGTC------------------------------- 9
---------------------------------------------TCCACGTAGATCGCGTCGTCGCC---------------------------- 1
----------------------------------------------CCACGTAGATCGCGTCGT-------------------------------- 3
-----------------------------------------------CACGTAGATCGCGTCGTCGCCG--------------------------- 2
----------------------------------------------------AGATCGCGTCGTCGCCGCC------------------------- 1
```

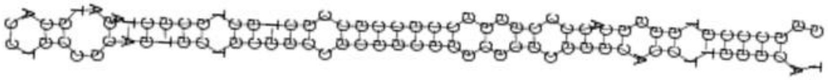

Supplement: S1 File — (PDF) [file pone.0151546.s001.pdf]
