# Supplementary material for: Identification and Analysis of Novel Viral and Host Dysregulated MicroRNAs in Variant Pseudorabies Virus-Infected PK15 Cells
Source: PLoS One. 2016 Mar 21;11(3):e0151546. doi: 10.1371/journal.pone.0151546 (PMC4801506; doi:10.1371/journal.pone.0151546)
Supplement: S2 File — (PDF) [file pone.0151546.s002.pdf]

S2 File. Expression levels detection of SC and Bartha-K61 encoded novel miRNAs using stem-loop qRT-PCR.

Figure A and B showed the expression levels of novel viral miRNA encoded from SC and Bartha-K61 strains, respectively. miRNAs expression levels were normalized to the U6 snRNA. Data are shown as the average ± standard deviation from three independent experiments.

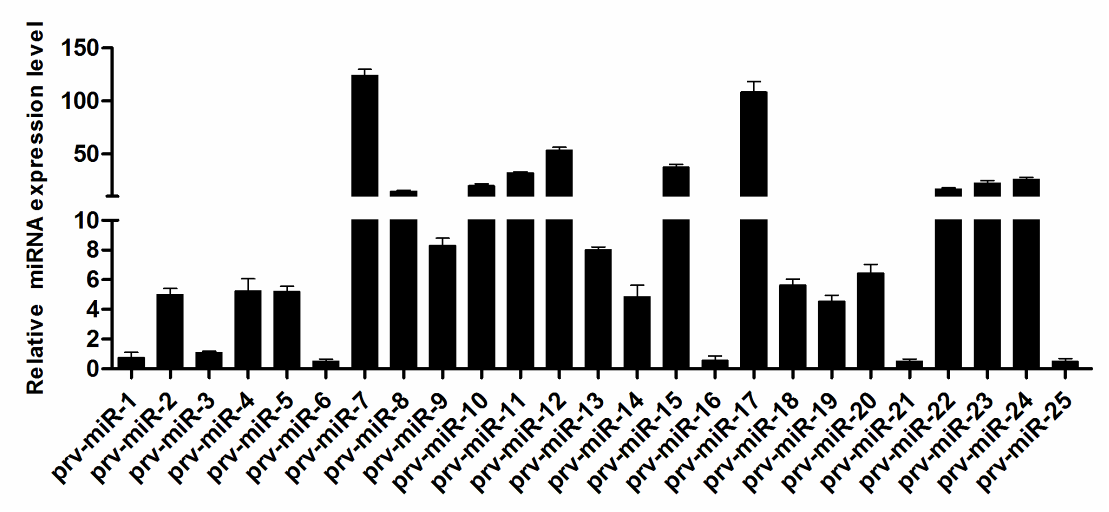

(A)

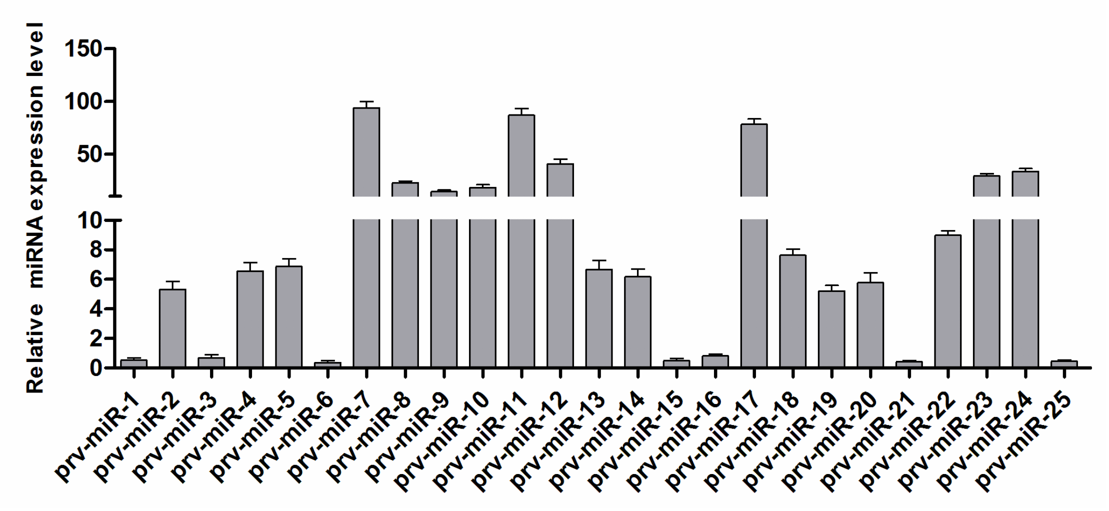

(B)
