# Supplementary material for: Identification and Analysis of Novel Viral and Host Dysregulated MicroRNAs in Variant Pseudorabies Virus-Infected PK15 Cells
Source: PLoS One. 2016 Mar 21;11(3):e0151546. doi: 10.1371/journal.pone.0151546 (PMC4801506; doi:10.1371/journal.pone.0151546)
Supplement: S1 Table — (DOCX) [file pone.0151546.s003.docx]

**S1 Table. Primers used to amplify virally and differentially expressed host miRNAs by stem-loop RT-qPCR.**

^a, b^ represents two kinds of primers with different stem loop structure, stem-loop RT-qPCR was performed using miRNA specific stem-loop RT primers together with corresponding miRNA specific forward (F) primersand universal reverse (UR) primer.

| **Primers** | **Sequence** |
| --- | --- |
| RT-prv-miR-1-5p^b^ | GTCGTATCCAGTGCAGGGTCCGAGGTATTCGCACTGGATACGACGCCCGACG |
| RT-prv-miR-2-3p^a^ | CTCAACTGGTGTCGTGGAGTCGGCAATTCAGTTGAGCCGAGAGG |
| RT-prv-miR-3-3p^a^ | CTCAACTGGTGTCGTGGAGTCGGCAATTCAGTTGAGTACAGCGC |
| RT-prv-miR-4-3p^a^ | CTCAACTGGTGTCGTGGAGTCGGCAATTCAGTTGAGGGCGCGCG |
| RT-prv-miR-5-3p^b^ | GTCGTATCCAGTGCAGGGTCCGAGGTATTCGCACTGGATACGACCGCGCC |
| RT-prv-miR-6-5p^b^ | GTCGTATCCAGTGCAGGGTCCGAGGTATTCGCACTGGATACGACACCTCC |
| RT-prv-miR-7-5p^a^ | CTCAACTGGTGTCGTGGAGTCGGCAATTCAGTTGAGGAGCGCGT |
| RT-prv-miR-8-3p^a^ | CTCAACTGGTGTCGTGGAGTCGGCAATTCAGTTGAGCGTGCGGG |
| RT-prv-miR-9-5p^b^ | GTCGTATCCAGTGCAGGGTCCGAGGTATTCGCACTGGATACGACCGTGGACG |
| RT-prv-miR-10-5p^a^ | CTCAACTGGTGTCGTGGAGTCGGCAATTCAGTTGAGCCGCCTCC |
| RT-prv-miR-11-5p^a^ | CTCAACTGGTGTCGTGGAGTCGGCAATTCAGTTGAGGGAGCCGG |
| RT-prv-miR-12-3p^a^ | CTCAACTGGTGTCGTGGAGTCGGCAATTCAGTTGAGCCCCCGCT |
| RT-prv-miR-13-5p^a^ | CTCAACTGGTGTCGTGGAGTCGGCAATTCAGTTGAGCATCGCCC |
| RT-prv-miR-14-5p^b^ | GTCGTATCCAGTGCAGGGTCCGAGGTATTCGCACTGGATACGACGGTTCACG |
| RT-prv-miR-15-5p^a^ | CTCAACTGGTGTCGTGGAGTCGGCAATTCAGTTGAGGCCCGGGG |
| RT-prv-miR-16-3p^b^ | GTCGTATCCAGTGCAGGGTCCGAGGTATTCGCACTGGATACGACTCCCTCCC |
| RT-prv-miR-17-5p ^a^ | CTCAACTGGTGTCGTGGAGTCGGCAATTCAGTTGAGCGCCGAGC |
| RT-prv-miR-18-3p^a^ | CTCAACTGGTGTCGTGGAGTCGGCAATTCAGTTGAG CCATCGCT |
| RT-prv-miR-19-5p^b^ | GTCGTATCCAGTGCAGGGTCCGAGGTATTCGCACTGGATACGACGCCGCGGC |
| RT-prv-miR-20-3p^b^ | GTCGTATCCAGTGCAGGGTCCGAGGTATTCGCACTGGATACGACTCGTCGAC |
| RT-prv-miR-21-3p^b^ | GTCGTATCCAGTGCAGGGTCCGAGGTATTCGCACTGGATACGACGGACGCCC |
| RT-prv-miR-22-3p^a^ | CTCAACTGGTGTCGTGGAGTCGGCAATTCAGTTGAGCGTCCGCA |
| RT-prv-miR-23-5p^b^ | GTCGTATCCAGTGCAGGGTCCGAGGTATTCGCACTGGATACGACCACGCCGA |
| RT-prv-miR-24-3p^b^ | GTCGTATCCAGTGCAGGGTCCGAGGTATTCGCACTGGATACGACCGTGCA |
| RT-prv-miR-25-5p^b^ | GTCGTATCCAGTGCAGGGTCCGAGGTATTCGCACTGGATACGACCCGGCGCC |
| RT-ssc-miR-10a-5p | GTCGTATCCAGTGCAGGGTCCGAGGTATTCGCACTGGATACGACACAAAT |
| RT-ssc-miR-132 | GTCGTATCCAGTGCAGGGTCCGAGGTATTCGCACTGGATACGACCGACCA |
| RT-ssc-miR-146b | GTCGTATCCAGTGCAGGGTCCGAGGTATTCGCACTGGATACGACGCCTAT |
| RT-ssc-miR-148a-3p | GTCGTATCCAGTGCAGGGTCCGAGGTATTCGCACTGGATACGACACAAAGT |
| RT-ssc-miR-199a-3p | GTCGTATCCAGTGCAGGGTCCGAGGTATTCGCACTGGATACGACTAACCAA |
| RT-ssc-miR-27a | GTCGTATCCAGTGCAGGGTCCGAGGTATTCGCACTGGATACGACGCGGAA |
| RT-ssc-miR-331-3p | GTCGTATCCAGTGCAGGGTCCGAGGTATTCGCACTGGATACGACTTCTAG |
| RT-ssc-miR-532-5p | GTCGTATCCAGTGCAGGGTCCGAGGTATTCGCACTGGATACGACACGGTC |
| prv-miR-1-5p^b^ (F)  prv-miR-2-3p^a^ (F) | GTTAGTGGCGGTCGGGGGGCG  TCGGCAGGCATGCACCTGTA |
| prv-miR-3-3p^a^ (F) | TCGGCAGGCGGCCAGCCCGGAC |
| prv-miR-4-3p^a^ (F) | TCGGCAGGCGACGACTGGGGG |
| prv-miR-5-3p^b^ (F) | CACTAGCGAGCTCTGCGACC |
| prv-miR-6-5p^b^ (F) | GTTAGTCGCAGGCGCGCGGCAT |
| prv-miR-7-5p^a^ (F) | TCGGCAGGTACCCGGCGCCCGTGA |
| prv-miR-8-3p^a^ (F) | TCGGCAGGCGAGCTCCTGCCGG |
| prv-miR-9-5p^b^ (F) | GTTAGTTACGCGGCGCGCTT |
| prv-miR-10-5p^a^ (F) | TCGGCAGGCCCGCGGACGCGCC |
| prv-miR-11-5p^a^ (F) | TCGGCAGGTCCACTTCTCGACGG |
| prv-miR-12-3p^a^ (F) | TCGGCAGGCCAATCGGGTGGC |
| prv-miR-13-5p^a^ (F) | TCGGCAGGCCGGGGAAGGGTC |
| prv-miR-14-5p^b^ (F) | GTTAGTCGCCTCGGGGCCGA |
| prv-miR-15-5p^a^ (F) | TCGGCAGGTCTCCGCCGAGACGA |
| prv-miR-16-3p^b^ (F) | GTTAGTCGCGGGCGGCGGGAG |
| prv-miR-17-5p ^a^ (F) | TCGGCAGGGCGACGGAAGGGGCAG |
| prv-miR-18-3p^a^ (F) | TCGGCAGGGGCCGGAACACCG |
| prv-miR-19-5p^b^ (F) | GTTAGTACGGAGCGCCTGGAC |
| prv-miR-20-3p^b^ (F) | GTTAGTCCGTGCTGGCCGTG |
| prv-miR-21-3p^b^ (F) | GTTAGTCACGCGGCGGGGGCGA |
| prv-miR-22-3p^a^ (F) | TCGGCAGGCCCGACGGGCTGG |
| prv-miR-23-5p^b^ (F) | GTCAGTCTGGATCGTGTGCC |
| prv-miR-24-3p^b^ (F) | GTTAGTCGTTGAGGGTCTGGA |
| prv-miR-25-5p^b^ (F) | ATTAGTACGGGCGCGCGGGC |
| ssc-miR-10a-5p (F) | GTTAGTTACCCTGTAGATCCGA |
| ssc-miR-132 (F) | GCGACATAACAGTCTACAGCCA |
| ssc-miR-146b (F) | GTTAGTTGAGAACTGAATTCC |
| ssc-miR-148a-3p (F) | GCGAGATCAGTGCACTACAGA |
| ssc-miR-199a-3p (F) | GTCCGTACAGTAGTCTGCACA |
| ssc-miR-27a (F) | GCGACATTCACAGTGGCTAAG |
| ssc-miR-331-3p (F) | TCTACTGCCCCTGGGCCTATC |
| ssc-miR-532-5p (F) | AGGTCCCATGCCTTGAGTGTAG |
| prv-UR^a^ | TCAACTGGTGTCGTGGAGTCGGC |
| prv-UR^b^ | TCCAGTGCAGGGTCCGAGGTAT |
| ssc-UR | TCCAGTGCAGGGTCCGAGGTAT |
| U6 (F) | CTCGCTTCGGCAGCACA |
| U6 (R) | GCGTGTCATCCTTGCGC |
