# Supplementary material for: Identification and Analysis of Novel Viral and Host Dysregulated MicroRNAs in Variant Pseudorabies Virus-Infected PK15 Cells
Source: PLoS One. 2016 Mar 21;11(3):e0151546. doi: 10.1371/journal.pone.0151546 (PMC4801506; doi:10.1371/journal.pone.0151546)
Supplement: S4 Table — (DOCX) [file pone.0151546.s006.docx]

**S4 Table. Novel porcine miRNA expressed in PK15 cells.**

The 239 mature sequence and sequence read count of novel porcine miRNAs predicted with miRDeep in each sequenced sample of PK15 cells.

| **mature sequence** | **mock** | **infected** |
| --- | --- | --- |
| AGACCCTGAGCTGCCTCTAGACT | 51 | 45 |
| AGAGGCTGGCCGTGATGAATTCG | 75 | 161 |
| GGTTGATCAGAGAACATACATT | 44 | 83 |
| GGACTTGGAGACCAGAGGACAGA | 79 | 95 |
| TCAGCCCCAGAGACAAGGAATCT | 192 | 253 |
| TTTGCTCTGCTCCTGCCACATG | 141 | 156 |
| TCCAGGGAAGAAAGGAGGAACA | 49 | 55 |
| TCCGTCGGGGGCCTGGCACA | 696 | 742 |
| ATAGAATCATATCTAGGACTGA | 171 | 220 |
| GTGTTGGTGTGCACTTATTT | 8123 | 5686 |
| GTGTGGAGAGAGTGGAAAGGTG | 307 | 407 |
| ACAGACCTGGGCATATATGTAT | 82 | 95 |
| ATCTGACCTTGCCGACCTCGCA | 21 | 52 |
| TTGGAGAAACAGGCTTTGGTGC | 62 | 63 |
| TGGCGGGAAACGGACCTGGCTG | 193 | 166 |
| TGTGAGGAGCCCTGGGAGACAGCA | 132 | 238 |
| GCAAAGCACACGGCCTGCAGAG | 135715 | 155920 |
| TCTGGGCAGAGGTAGACAGGGTGG | 212 | 312 |
| CCAGGGGGCTGACACGGAGGAAG | 369 | 491 |
| GTGGGACCTAGGGCTGCGGCTG | 74 | 85 |
| GCCGGAGGCGGGGCTACCTGGG | 50 | 48 |
| GCTGGAGGTGGGCTGTACAGAT | 124 | 143 |
| TGGGGAGGGCCGGACAGAGCAG | 249 | 294 |
| GGGACTGAGCATGGAGCGGAG | 47 | 69 |
| TGGTGAGGAGCTGAGCCGGGCT | 88 | 50 |
| CTTCGTGGTCAGCTCCCTTT | 145 | 195 |
| TGGCAGTGTATTGTTAGCTGGT | 241 | 346 |
| GTCACTGAGGCGGACAGGCAGG | 55 | 65 |
| CACGCTTGTGTCGTTGGAGTGG | 185 | 231 |
| GTGTGGGGACGAGCCGGCTCT | 84 | 231 |
| AGCGAGGAACTGAGGAAGGTATG | 161 | 169 |
| AACCTTGCTGGAGATCTTGGA | 295 | 259 |
| TGCCGGGCGGGAGCACGTGGCT | 84 | 103 |
| TCCTGGAGGACGTGCTGTGC | 629 | 638 |
| TCGGGGTGTGAGATGTGCGCGT | 223 | 241 |
| TGTGGAGACCTCAGCACGTAG | 49 | 67 |
| AGGGACGTGATTTGGAAAGAGA | 55 | 68 |
| TGGACGGTGCTTAGAGAGAGA | 104 | 129 |
| AGAGGAGCAGCGTGGACCCACCT | 244 | 326 |
| AGGGAAGGTGGGGTGAGGGAGG | 68 | 81 |
| TTCCGGAGCAAGTTGTGGAGG | 51 | 44 |
| TAGTGCAATATTGCTTATAGGGTTT | 378 | 353 |
| GTCACTGAGGCGGACAGGCAGG | 55 | 65 |
| CGGGAGCCTGCGGCTGGGCCGG | 42 | 51 |
| TTACAGTATTAGTCGCTTTT | 1104 | 1774 |
| TATCCCCATGGAGTCTGTTGCC | 79 | 71 |
| AGGGGGAGAGAGCTGCGCTCAGA | 179 | 177 |
| AGGGAAGGTGGGGTGAGGGAGG | 68 | 81 |
| ACAGGACGAGGGGATTGCGCTGT | 149 | 153 |
| GCAAGATGATGGCATTCTGACC | 1170 | 1088 |
| AAGGAAGGGAGACGGACCCAAG | 50 | 41 |
| TGGACGGGAAGAGGAGGGGCTC | 40 | 48 |
| TTTGCTCTGCTCCTGCCACATG | 141 | 156 |
| TAGAGTGCTGGCCTGGGGGGTG | 85 | 61 |
| ACGGCGGGAGTGGAGTCGGCG | 71 | 64 |
| TTTGGTTTGTTTGGGTTTGTT | 50 | 49 |
| AGGAGCAGGAGTCTGGGCTGA | 2037 | 2294 |
| GAGAGATCAGAGGCGCAGAGT | 343652 | 436921 |
| TTCAAGTAACCCAGGATAGGCT | 13965 | 11668 |
| TGGAGAGAGTTGTGGAAGGTGC | 47 | 80 |
| TGGGTGAGAGCACAGCAGAACT | 120 | 113 |
| CTCGGAGGCTCCATCATGGCGT | 332 | 392 |
| CACGGTCCCTCCGCGAGCACCGG | 82 | 101 |
| TGCTGACTGACCTCCAGGGCAT | 138 | 137 |
| CAGGGACAGGGGCACAGGTGCT | 32 | 55 |
| AAGGAGCTGAGGACGGAGAAGGA | 6273 | 10755 |
| AGGGCGCCAGGCAGGACCGCTTT | 161 | 259 |
| TGGGTTGAATGGCTGGTTCTGA | 79 | 80 |
| GGGATTCTCGGGGGGCTGGTT | 41 | 54 |
| AGGGAGACGGCAGACAAGAGCG | 37 | 55 |
| GCAACAGTAGGGGGCCGTAGCG | 1292 | 1490 |
| GAGGTGCTGCAGGAGGTGGGCTCT | 3413 | 4081 |
| ATGTGCTCGACGGATTTGGTGT | 95 | 99 |
| GAGGTGCTGCAGGAGGTGGGCTCT | 3413 | 4081 |
| ATGTGCTCGACGGATTTGGTGT | 95 | 99 |
| GATTTGGCTGGCTGGCTGAAGGCA | 47 | 69 |
| AAGCGGGGCTGGGAAGAGGTGA | 46 | 82 |
| AGACCCTGAGCTGCCTCTAGACT | 51 | 45 |
| ACTGGAACATGGCAGAGGACA | 51 | 55 |
| ACCTCCGCCGGTACTGCGCGCA | 72 | 86 |
| TGTAATGATGGAATACTGAAA | 479 | 404 |
| TTTGTTCGTTCGGCTCGCGTGA | 211 | 693 |
| GGGACTGAGCATGGAGCGGAG | 47 | 69 |
| TTCTGGAGAAGAAGAGGTCTT | 114 | 127 |
| AGGCAGTGTATTGTTAGCTGGCT | 102 | 83 |
| AAAGATGCATGTGTGGGATACC | 96 | 66 |
| CCCGTGGAGGATGAGCTGGCT | 59 | 35 |
| AGTGTGCAGGGACAGCCTCGGG | 286 | 336 |
| CTCAGAGAGGTGGCAACAGACA | 32 | 51 |
| TAGGTGTAGAAGTTAAGGAGGCC | 57 | 62 |
| AGATGAACAGCTGTATACCTAT | 176 | 219 |
| TCCGGGGTAAAATGGCGGCTTT | 34 | 58 |
| GAGGCGAGGTGGCTCACGCGTT | 190 | 244 |
| TGGGGACGGAGAGAAGTGGGTG | 51 | 54 |
| ATTGGCACAATGAGAGGAACAGTT | 487 | 451 |
| TGCCCCGTCGAAGCTGTCGGTG | 311 | 323 |
| AAGGAAGGGAAGGAGGAGGATG | 82 | 62 |
| TCTGACTCCAGAGCTTCAGACA | 66 | 42 |
| TGAACAGAGGTCATGAACAGAGC | 104 | 141 |
| GCTAGGGAGCGGGAAGAGGTGG | 164 | 218 |
| TGGACTAACTGTGGTATTGGGA | 129 | 128 |
| AGTCTGACTATGTAGGGGCCT | 108 | 101 |
| AGACCGGGGACCGGAGCGAGCAGA | 327 | 403 |
| AGAATTGCGTTTGGACAATCAGT | 141 | 152 |
| TCCCACGGGGGTCGCCGCCATC | 37 | 60 |
| ACCTGGAAGAGGCGGGACTTT | 6 | 73 |
| CAGAGCTGTTTGTGAACTGCAGGT | 291 | 201 |
| CGGCGGCTGCGGGGAGACCCCG | 272 | 368 |
| TGGTGAGGAGCTGAGCCGGGCT | 88 | 50 |
| ACAGTCAACGGTCGGTGGTTT | 1729 | 1941 |
| CGCGTACCAAAAGTAATAATGTC | 85 | 109 |
| AAGGGGACGGGGAGAAGGGAGA | 78 | 131 |
| GACCTGTGATGTCGTTTCTGAGA | 125 | 92 |
| TCGGACTCAGGACGGGCCTTGA | 123 | 153 |
| GGGCAAGCCTGCGGAGGTGTGG | 364 | 523 |
| GACTGCACTGCTGGGCCTGGCA | 64 | 68 |
| TGGGGGACTGTGAACAGCGGG | 1942 | 2445 |
| GTAGGGCGGGCTGCACCGGGCA | 48 | 53 |
| CTCTGCCCGCTCTCTGTCTTACA | 119 | 158 |
| AGCTGGGACTGAAGGTGGGCGC | 51 | 48 |
| GCCGGCAGTGCTGTAGTTGGTCT | 104 | 89 |
| CTTCGTGGTCAGCTCCCTTT | 145 | 195 |
| ACTGACAGGAGAGCATTTTAA | 79 | 108 |
| TAAGGGCAAAGTAGGGACAGCT | 892 | 1449 |
| AAGGGGGAACAAGGAGGACAAGA | 52 | 84 |
| TCTGCACATGTAGATACACGCAG | 127 | 144 |
| GTGGGAGCAGGTGGGTAGGCG | 63 | 89 |
| AAGAGACCGGGGCCACAAGAGC | 85 | 117 |
| AGCAGGTTTTGGGCCCCTGGA | 26 | 61 |
| TGGGAAGGGGCACTGAGAGGTCT | 158 | 196 |
| CAGGGCGGGTGGGGAGGCTG | 58 | 48 |
| GCAGAGTGACGTGGCTGGAAGT | 91 | 113 |
| TCACCGACCGCCGGGCGGCAGGC | 47 | 50 |
| ACCGATGCCTGGGCCGTCTGG | 44 | 54 |
| TAATTTTATGTATAAGCTAGT | 828 | 922 |
| TGCCTCCCTTGAGCAGCGGACTG | 44 | 68 |
| TGGGGAGGGCATTGTGGTGACG | 39 | 53 |
| GTTCTGAGACGGACATCTGGT | 60 | 56 |
| TGCTAGGGCTCGCGGAACGCGGT | 76 | 92 |
| TGGACCCAGGAGAACGGCTCTCG | 72 | 111 |
| CAGACCAGCAGGATGTGGACT | 234 | 284 |
| CCTTGGTTATGGCAGAATGTT | 49 | 53 |
| ATGGGTCTGACGGAGCAGCCGC | 941 | 751 |
| GGTTCGGCTGTGCGGGCACT | 673 | 952 |
| ACCGATGCCTGGGCCGTCTGG | 44 | 54 |
| GTAGGTGGGATGCAGGGGAGC | 394 | 589 |
| TGGCTCTGCGAGGTCGGCTCA | 43997 | 44358 |
| TCTGGTTCAGGGTACATGGCACT | 154 | 212 |
| TCTGGAGCAGCAGGAGGACTAT | 48 | 51 |
| GATATGAGAGTGTTGGTCCTGA | 452 | 391 |
| TCCTTGGAGCCTGCTGGAGT | 47 | 57 |
| ATGGGTCTGACGGAGCAGCCGC | 942 | 751 |
| AGGCCGCGGCAGCTGTGGGTG | 329 | 330 |
| TCCGTGGAGGAAGATGCTGTGA | 263 | 202 |
| AGGAGAAAGCAGACGGGCTGCA | 1998 | 2843 |
| AGGGGATCTTCAGGGTGGTGACG | 81 | 124 |
| AGAGGAGGAAAACAGAGCCAGG | 60 | 105 |
| AGGTTAGGGTGAGTGGCTGTTT | 289 | 302 |
| TTGGGGAATGAAGAAGAGTCAGCA | 50 | 43 |
| AGGAGACAGGTGCTAAGAGCTCT | 702 | 944 |
| AATCGTGGTGCTGGAACCCGGCT | 262 | 297 |
| TCCGTGGAGGAAGATGCTGTGA | 263 | 202 |
| TGAGACTGGGATGACATGTTT | 160 | 161 |
| CAGGACCAGGTGAGCGCCAGC | 141 | 114 |
| AAGAAGAGGACAACTGTTGGAAC | 54 | 58 |
| GAAGCATGGAGAGGCCTGGGACA | 81 | 125 |
| GCGGACCCAAAGGCGGAGAGGTG | 54 | 55 |
| TGGACCTTCAGCATTTCTCCCT | 171 | 155 |
| CGGCTCTGGGTCTGTGGGGAG | 3761 | 3701 |
| CATGTCCGCGGGTTCCCTATC | 199 | 188 |
| CAACACTGTGCTGGAAGATGGA | 3899 | 5199 |
| TCTGGGACTGAAGGCCGCGGCC | 150 | 102 |
| TGGAGAGCAGGATGTAGCCAAA | 45 | 54 |
| AGACTGGCGGGGCCCTCGGCC | 59 | 42 |
| TCTGGAGGCAGCGGCTGGCTCT | 253 | 293 |
| TTGAACCTGATAGCAGCTGCCG | 687 | 937 |
| TGCGTTCATTCTGTTTGGCCTG | 148 | 109 |
| CCGGAAGTGGAAGTAAGCGCAGGT | 347 | 322 |
| TAATACTGCCGGGTAATGATGGA | 31 | 102 |
| TTCGCGGCTCCCTGGACCAGCGCA | 68 | 48 |
| TTTGGTTTGTTTGGGTTTGTT | 58 | 55 |
| AGGCGGGCGCCGGGCTCGTGAGG | 434 | 487 |
| ACGCAGGAGACGTGGAGCGCTT | 114 | 114 |
| AGCAGCGAGGCCGTAGCTTGGT | 175 | 151 |
| CGGGCTGCGTGTCCCTCGGCAG | 200 | 212 |
| AGGAGGGAAGGCGGATGAGAGCT | 819 | 1022 |
| TCTGCACCGGGGCTTTGAGGGG | 19 | 44 |
| TCGGGGAGGCTGTGCAGCGCGG | 793 | 1074 |
| TCATGATATAGAGGTAAATAGT | 2842 | 2466 |
| CTGGGGAGCAGGTAGGGATGGCGT | 102 | 148 |
| GACGCGGTGGTAGGAGGTGGA | 145 | 169 |
| TGAGCGGCTGTGGAGCGGCGTT | 98 | 87 |
| TGTGTGTGTGGGCGCCGGACG | 26916 | 37677 |
| TGGGTGGAGGAAAGCGGGGCCA | 261 | 370 |
| TCCAGGGTGGAAAAGAGCTTG | 55 | 71 |
| TCTTTTGTTTGATTGTTTGGGTTT | 83 | 63 |
| TCGGGAGCAGAACCTCACGTCAT | 37 | 64 |
| TAGCGGGGATGACAAGTGTGGC | 48 | 83 |
| TTGGGGAAGGGGGGAGTTCAGT | 105 | 116 |
| TCAGTCAAGGGAGCTGGCACTCA | 70 | 52 |
| TGGTTGGGAAGGAAGAAGCTGC | 253 | 293 |
| CTGGGGAGCAGGTAGGGATGGCGT | 102 | 148 |
| GACGCGGTGGTAGGAGGTGGA | 145 | 169 |
| TGCAGGTGGAGCCCAGGGGAAC | 253 | 319 |
| TGTCATGCTGGGGAGTGTAGTGA | 45 | 60 |
| ATGAGATGCAGAGCTTGGATG | 52 | 31 |
| GAGGGGCACACACATGAGCAGTG | 161 | 139 |
| TAGAGGGAGGGAAAGAGAAGAGGT | 59 | 110 |
| GGCGGGGCGGCAGTTCCTGGAGG | 194 | 202 |
| GAAGGACACGGGAGTATTTT | 126 | 147 |
| AAAGGATGGATTGGACAGGCCT | 128 | 129 |
| GAGGGGCACACACATGAGCAGTG | 161 | 139 |
| TGGGGGATGATAGCAAAGACAA | 73 | 83 |
| TGTGGAGCACGTAGAAGGAAGA | 899 | 1237 |
| CGGGGCGGGCGGCAGCGGGCG | 57 | 46 |
| TGGGTTCTGCGGAGTGGAGCC | 55 | 40 |
| TATGGCTTTTCATTCCTATGTGA | 651 | 747 |
| TCTGGCGGGAAGTTGTGGTCC | 5169 | 7612 |
| CACAGCAAGTGTAGACAGGCA | 1056 | 1366 |
| AGGTAGTCTGAACACTGGGGCGA | 97 | 74 |
| CCTGTCAGAAGGTCGGGTAGCA | 205 | 224 |
| CACGCTTGTGTCGTTGGAGTGG | 185 | 231 |
| TCGTGCACAGATGTGGTCTCGG | 44 | 93 |
| CCGACACAGGTTGGAGAGGCTCG | 70 | 49 |
| TATGGCTTTTCATTCCTATGTGA | 651 | 747 |
| TCACTGGAGTTTTGTTTCAACATT | 221 | 196 |
| GAGAGCACTGGGGCAGCTGCAG | 67 | 99 |
| TCTGTACGTGGGAGGGTGTGT | 202 | 369 |
| GTGTGGGGACGAGCCGGCTCT | 84 | 91 |
| TTGGAGGTGGTGGAGGCAACT | 56 | 48 |
| CATCCCTTGCATGGTGGAGGGT | 161 | 194 |
| GTGCACCTGGGCAAGGATTCTGA | 91 | 95 |
| TACCCATTGCATATCGGAGTTG | 6289 | 5689 |
| TACCCAGAGCGTGCAGTGTGAA | 227 | 251 |
| TGCACGGAGCAGCAGGGTCTGA | 250 | 276 |
| TGACCAGGCAGGTGCTGTTCTCT | 491 | 352 |
| TGAACGGCGCCTGTGTGGTTAGA | 141 | 135 |
| AATGGCGCTTTTTTGTGAAGA | 222 | 241 |
| TACTGCAGAGAGTGGCAATCAT | 18255 | 22125 |
